# ApicoAlign: an alignment and sequence search tool for apicomplexan proteins

**DOI:** 10.1186/1471-2164-12-S3-S6

**Published:** 2011-11-30

**Authors:** Jamshaid Ali, Umadevi Paila, Akash Ranjan

**Affiliations:** 1 Computational and Functional Genomics Group, Centre for DNA Fingerprinting and Diagnostics, A Sun Centre of Excellence in Medical Bioinformatics, Hyderabad 500001, India

## Abstract

**Background:**

Over the recent years, a number of genomes have been successfully sequenced and this was followed by genome annotation projects to help understand the biological capabilities of newly sequenced genomes. To improve the annotation of *Plasmodium falciparum* proteins, we earlier developed parasite specific matrices (PfSSM) and demonstrated their (Smat80 and PfFSmat60) better performance over standard matrices (BLOSUM and PAM). Here we extend that study to nine apicomplexan species other than *P. falciparum* and develop a web application ApicoAlign for improving the annotation of apicomplexan proteins.

**Results:**

The SMAT80 and PfFSmat60 matrices perform better for apicomplexan proteins compared to BLOSUM in detecting the orthologs and improving the alignment of these proteins with their potential orthologs respectively. Database searches against non-redundant (nr) database have shown that SMAT80 gives superior performance compared to BLOSUM series in terms of E-values, bit scores, percent identity, alignment length and mismatches for most of the apicomplexan proteins studied here. Using these matrices, we were able to find orthologs for rhomboid proteases of *P. berghei*, *P. falciparum* &*P. vivax* and large subunit of U2 snRNP auxiliary factor of *Cryptosporidium parvum* in *Arabidopsis thaliana*. We also show improved pairwise alignments of proteins from Apicomplexa viz. *Cryptosporidium parvum* and *P. falciparum* with their orthologs from other species using the PfFSmat60 matrix.

**Conclusions:**

The SMAT80 and PfFSmat60 substitution matrices perform better for apicomplexan proteins compared to BLOSUM series. Since they can be helpful in improving the annotation of apicomplexan genomes and their functional characterization, we have developed a web server ApicoAlign for finding orthologs and aligning apicomplexan proteins.

## Background

One of the important goals of post-genomic era is to develop tools/services to help in the annotation of hypothetical/putative proteins of newly sequenced genomes. In case of *Plasmodium falciparum*, approximately ~60% of its genes did not show sequence similarity to known genes [1]. This organism showed an unusual amino acid composition and substitution in its proteins due to its extreme AT rich genome composition [2,3]. As a result, many proteins show no or low sequence match to the known proteins in the database, posing a major difficulty in genome annotation. In order to address this issue we developed the symmetric Smat series and the asymmetric PfFSmat60 and demonstrated their better performance over standard matrices (BLOSUM and PAM) [2]. Here we extend the use of these matrices to better annotate the proteins of other apicomplexa like *Plasmodium berghei*, *Plasmodium chabaudi*, *Plasmodium knowlesi*, *Plasmodium vivax*, *Plasmodium yoelii yoelii*, *Toxoplasma gondii*, *Cryptosporidium parvum*, *Theileria parva* and *Neospora caninum*. After benchmarking the performance of these matrices for apicomplexan proteins, we develop ApicoAlign a web server for finding orthologs and aligning apicomplexan proteins using a novel series of matrices.

## Implementation

ApicoAlign is a web-based application written in Perl/CGI language. The web server has five applications (1) Search Database (2) Search a genome (3) Reciprocal Hit (4) Best Bidirectional Hit and (5) Pairwise Alignment for apicomplexan proteins. The sample input buttons have been provided for some apicomplexan species for automatic loading of sample protein sequences in the required fields for each option. The parasite specific symmetric matrices (Smat series) consisting of Smat50, Smat60, Smat70, Smat80 and Smat90 are provided for first four applications. Smat matrices have been earlier demonstrated to work best for database searches [2] of *P. falciparum* and here we show their superior performance for other apicomplexa to increase the utility of these matrices. For comparison, the standard BLOSUM62 matrix and similar entropy matrix BLOSUM90 have been provided in the drop down menu. For the first four applications, the default values for gap open and extension penalties have been set to 10 and 1 respectively that are defined best for the standard matrices with entropy similar to Smat series. Few other combinations of gap open and extensions have also been provided that the user can try. E-value cut-off may be defined by the user.

### Search database

The non-redundant (nr), swiss-prot and PDB databases have been provided for finding orthologs for apicomplexan proteins using parasite specific and standard matrices. The input should be a single protein sequence in FASTA format which can be pasted in the text box provided or uploaded through a file.

### Search a genome

This option has been provided for finding hits for apicomplexan proteins across different genomes provided in the drop down menu. The input is protein sequences in FASTA format which can be pasted in the text box or uploaded through a file (upto 5 MB).

### Reciprocal hits

This option has been provided for finding reciprocal hits for apicomplexan proteins across different genomes provided in the drop down menu. The input is protein sequences in FASTA format which can be pasted in the text box or uploaded through a file (upto 5 MB).

### Best Bidirectional Hit

The method of BBH (Bidirectional Best Hit) [4] has been employed for the search of potential orthologs of apicomplexan proteins across a range of organisms. The input for bidirectional ortholog detection is a protein sequence file of the query genome and that of the subject in the fasta format. The subject proteome may be either selected from the list of the organisms provided in the web page or in case of a user specific sequence file it may be uploaded through the file upload option. Large sequence files may take a longer run time and the size of the uploaded query and subject sequence files is limited to 25 MB.

### Pairwise alignment

The pair-wise alignment option uses the water program (EMBOSS package, version 6.3.1) [5] for performing local alignments of the apicomplexan query protein and its potential ortholog. The asymmetric parasite specific matrix, PfFSmat60 is provided for performing these alignments along with standard matrices EBLOSUM62, EBLOSUM90, EPAM200, and PfFSmat60. PfFSmat60 has been demonstrated to perform best for pair-wise alignments [2], where the alignments span motif like regions of the protein. PfFSmat60 is a scaled version of a unique asymmetric matrix [2] used here for improving the alignment of an apicomplexan protein with its strongly suspected ortholog. Hence, users are not encouraged to use this matrix indiscriminately for non-orthologous proteins. The input is a single protein sequence in fasta format for query as well as subject. The user may provide (or use default values of) the gap open and extension penalties for the pair-wise alignment. PfFSmat60 was developed in context of *Plasmodium falciparum* and represents unidirectional substitutions [2] whose usage we extend to other apicomplexans in this study. Hence, one of the limitations of the pairwise alignment is that the query sequence is restricted only to apicomplexa, therefore, the query and subject proteins should not be reversed in their order.

## Results and discussion

To check whether *Plasmodium falciparum* Specific Substitution Matrices (SMAT and PfFSmat) perform better for other apicomplexan species, we carried out database searches against non-redundant database (nr) and found best bidirectional hits across different bacterial and eukaryotic genomes using BLOSUM and SMAT series of matrices.

### Amino acid composition of different apicomplexan species

**Figure 1:**
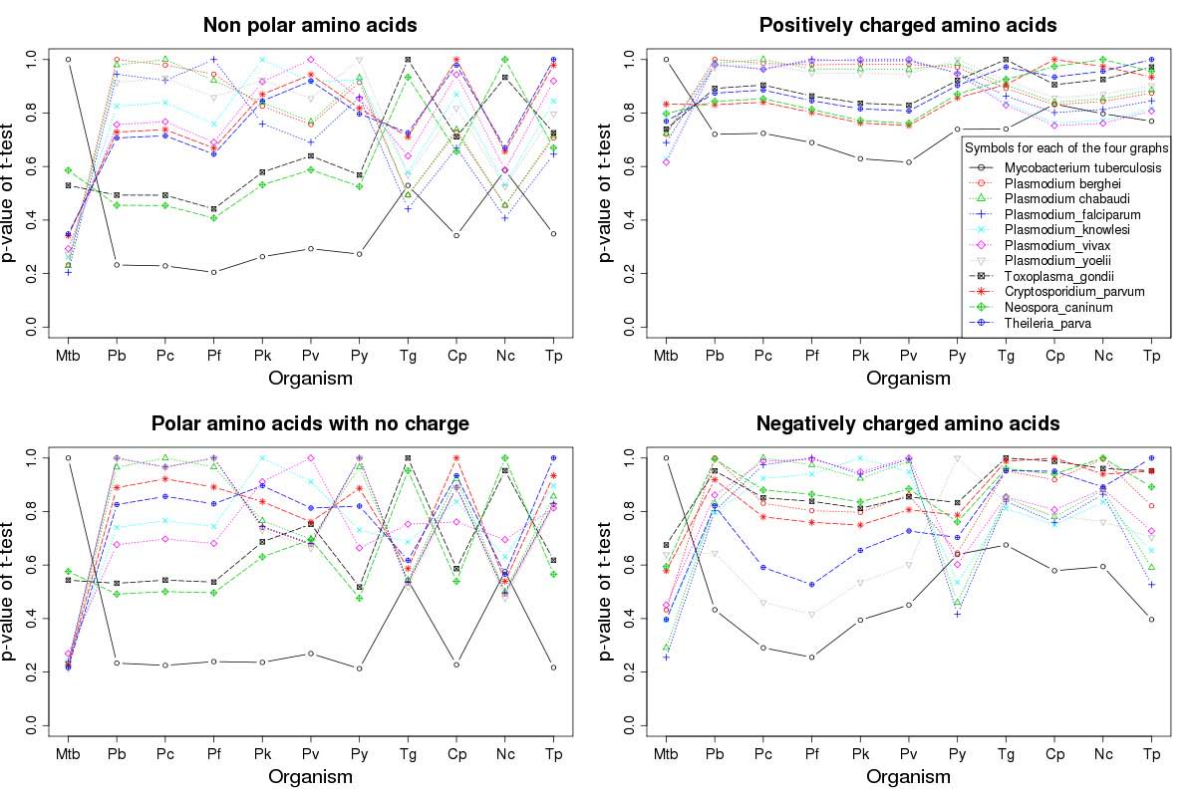
**Different apicomplexan genomes show similar amino acid composition.** Amino acid compositions were calculated for ten apicomplexan and one non-apicomplexan species (the labels on X-axis: Mtb for *Mycobacterium tuberculosis*, Pb for *Plasmodium berghei*, Pc for *Plasmodium chabaudi*, Pf for *Plasmodium falciparum*, Pk for *Plasmodium knowlesi*, Pv for *Plasmodium vivax*, Py for *Plasmodium yoelii yoelii*, Tg for *Toxoplasma gondii*, Cp for *Cryptosporidium parvum*, Nc for *Neospora caninum* and Tp for *Theileria parva*). Each point of graph represents the p-value between two genomes: one corresponding to X-axis and the other corresponding to colour and style of that particular line. The lines in each of the four graphs represent different genomes in the order shown in the legend of topright graph.

In our earlier study, we have shown that *Plasmodium falciparum* has biased amino acid choices for its proteins and this is one of the reasons that standard matrices BLOSUM & PAM do not perform well in this case [2]. Since the SMAT and PfFSmat60 matrices were originally developed for *Plasmodium falciparum*, we calculated the amino acid composition for all the proteins of apicomplexan genomes and compared them with that of non-apicomplexan *Mycobacterium tuberculosis* genome (details of calculation in Methods section). The amino acids were divided in four categories based on their properties: (a) non polar, (b) polar with no charge, (c) positively and (d) negatively charged amino acids. The p-value (t-test) between two genomes was calculated by taking means of amino acids of a particular category at a time and also for individual amino acids. The higher the p-value, the closer are the two genomes in terms of amino acid composition. Figure 1 shows that all the apicomplexan genomes are having low p-values when compared to *Mycobacterium tuberculosis* while they have higher p-values when compared to each other. In case of non polar amino acids 8 out of 10 apicomplexan species (except *Toxoplasma gondii* &*Neospora caninum*) show minimum p-value with *Mycobacterium tuberculosis* while in case of positively charged amino acids all apicomplexan species except *Cryptosporidium parvum* &*Neospora caninum* show minimum p-value with *Mycobacterium tuberculosis*. We observed similar patterns for polar amino acids with no charge (two exceptions *Toxoplasma gondii* &*Neospora caninum*) and negatively charged amino acids (only one exception *Plasmodium yoelii yoelii*). We also calculated the p-values between genomes using individual amino acid fractions. The amino acid compositional similarities (with *Plasmodium falciparum*) for *Mycobacterium tuberculosis*, *Plasmodium berghei*, *Plasmodium chabaudi*, *Plasmodium knowlesi*, *Plasmodium vivax*, *Plasmodium yoelii yoelii*, *Toxoplasma gondii*, *Cryptosporidium parvum*, *Neospora caninum* and *Theileria parva* were 20%, 100%, 100%, 75%, 85%, 100%, 40%, 45%, 40% and 50% respectively when amino acids were considered individually rather than groups (Additional File 1: Supplementary Table S1). The amino acid composition of *Plasmodium falciparum* proteins is quite similar to that of other apicomplexan species and within apicomplexan species the similarity is more with other *Plasmodium* sps. As different apicomplexan genomes show similar amino acid composition (to a good extent) as that of *Plasmodium falciparum*, therefore substitution matrices (SMAT & PfFSmat series) originally made for *Plasmodium falciparum* should perform better (when compared to BLOSUM & PAM) for other apicomplexan species also.

### Database searches

**Figure 2:**
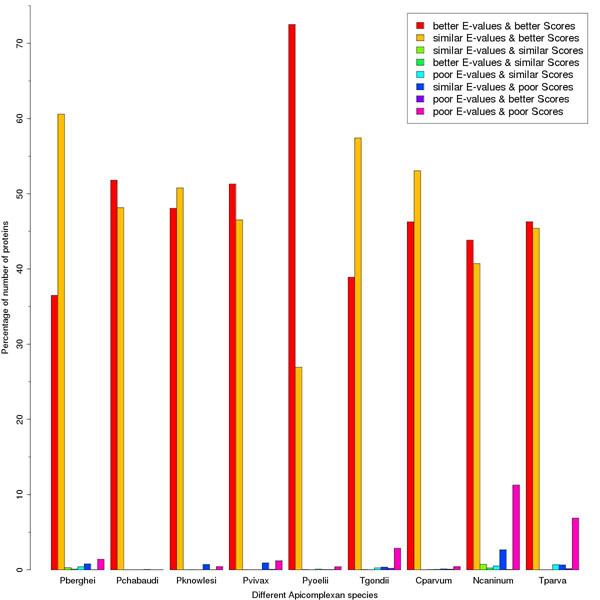
**Comparison of E-values & bit scores given by SMAT80 and BLOSUM90 matrices.** BLAST searches were performed against non-redundant (nr) database for nine Apicomplexan species (the labels on X-axis: Pberghei for *Plasmodium berghei*, Pchabaudi for *Plasmodium chabaudi*, Pknowlesi for *Plasmodium knowlesi*, Pvivax for *Plasmodium vivax*, Pyoelii for *Plasmodium yoelii yoelii*, Tgondii for *Toxoplasma gondii*, Cparvum for *Cryptosporidium parvum*, Ncaninum for *Neospora caninum* and Tparva for *Theileria parva*) using SMAT80 and BLOSUM90 matrix. The best non-self hits common to both matrices from these BLAST results were divided in eight categories shown in the legend at topright position of figure. The percentage for each category was calculated and it was observed that most of the apicomplexan proteins fall in first two categories that means most of apicomplexan proteins give better or similar E-values and better bit scores with SMAT80 compared to BLOSUM90 matrix.

**Figure 3:**
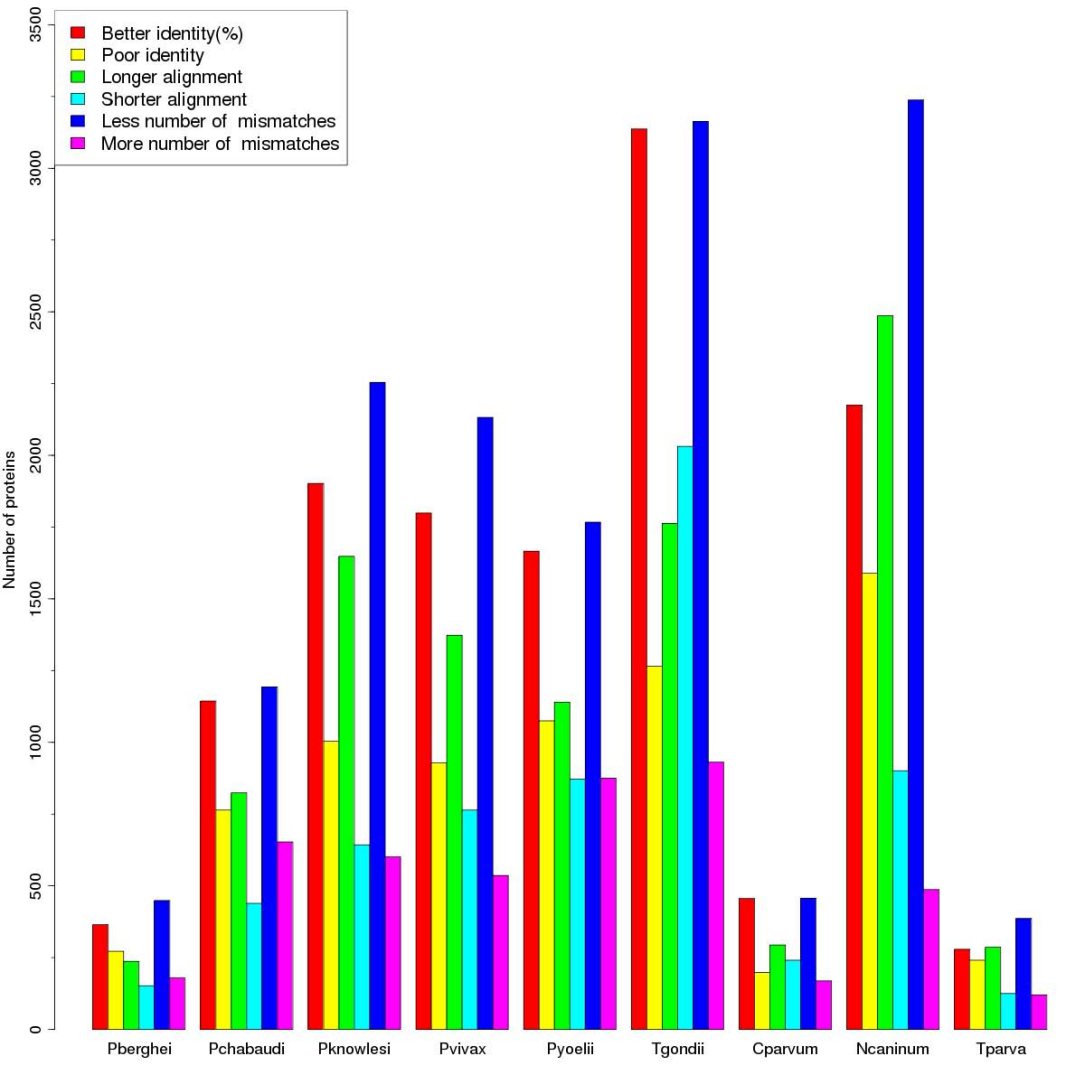
**Comparison of percent identity, alignment length and mismatches given by SMAT80 and BLOSUM90 matrices.** BLAST searches were performed against non-redundant (nr) database for nine Apicomplexan species (the labels on X-axis: Pberghei for *Plasmodium berghei*, Pchabaudi for *Plasmodium chabaudi*, Pknowlesi for *Plasmodium knowlesi*, Pvivax for *Plasmodium vivax*, Pyoelii for *Plasmodium yoelii yoelii*, Tgondii for *Toxoplasma gondii*, Cparvum for *Cryptosporidium parvum*, Ncaninum for *Neospora caninum* and Tparva for *Theileria parva*) using SMAT80 and BLOSUM90 matrix. The best non-self hits common to both matrices were filtered out from these BLAST results. The percent identity, alignment length and number of mismatches were divided in two categories- better or poor using SMAT80 compared to BLOSUM90 and the numbers of proteins for these categories were calculated. We see here a more number of proteins belonging to better category in each case.

Database searches (BLAST) were performed for all the proteins of nine apicomplexan species (*Plasmodium berghei*, *Plasmodium chabaudi*, *Plasmodium knowlesi*, *Plasmodium vivax*, *Plasmodium yoelii yoelii*, *Toxoplasma gondii*, *Cryptosporidium parvum*, *Neospora caninum* and *Theileria parva*) using SMAT80 and BLOSUM90 matrices against non-redundant (nr) database. The identical hits (best non-self hits) given by SMAT80 and BLOSUM90 were compared for the improvement in E-values and bit scores. *Plasmodium falciparum* has been omitted as we have already reported the results for it [2]. These hits were classified in eight categories (1) better E-values and better scores with SMAT80 compared to BLOSUM90, (2) similar E-values and better scores, (3) similar E-values and similar scores, (4) better E-values and similar scores, (5) poor E-values and similar scores, (6) similar E-values and poor scores, (7) poor E-values and better scores and (8) poor E-values and poor scores. The percentage of proteins was calculated for each category for all the nine apicomplexan species studied in this paper (Figure 2). The best non-self hits common to SMAT80 and BLOSUM90 matrices along with their E-values and bits scores for these nine apicomplexan species against non-redundant (nr) database have been provided as Additional File 2: Supplementary Table S2. Similarly the performance of SMAT80 was compared with that of BOSUM62 matrix (Additional File 3: Supplementary Figure 1). After observing improvement in E-values and bit scores, we compared how many proteins give better or poor percent identity, longer or shorter alignment length and less or more number of mismatches using SMAT80 compared to BLOSUM90 (Figure 3) and BLOSUM62 (Additional File 4: Supplementary Figure 2). In *Plasmodium berghei*, SMAT80 matrix (when compared to BLOSUM62) gives better & poor percent identity for 1066 & 698 proteins respectively, longer & shorter alignment for 673 & 426 proteins respectively and less & more number of mismatches for 1092 & 614 proteins respectively. Similarly, SMAT80 matrix performs better for other apicomplexan species studied here (Figure 3 and Additional File 4: Supplementary Figure 2). However in the case of *Toxoplasma gondii*, 2096 proteins give shorter alignment while 1833 proteins give longer alignment with SMAT80 matrix (compared to BLOSUM62) probably due to the large increase in percent identity (3247 proteins give better percent identity while 1264 proteins give poor percent identity). Therefore, these comparisons of SMAT80 with BLOSUM90 and BLOSUM62 clearly show the superior performance of SMAT80 matrix over BLOSUM62 & BLOSUM90 for most of the apicomplexan proteins.

### Best Bidirectional Hits

**Figure 4:**
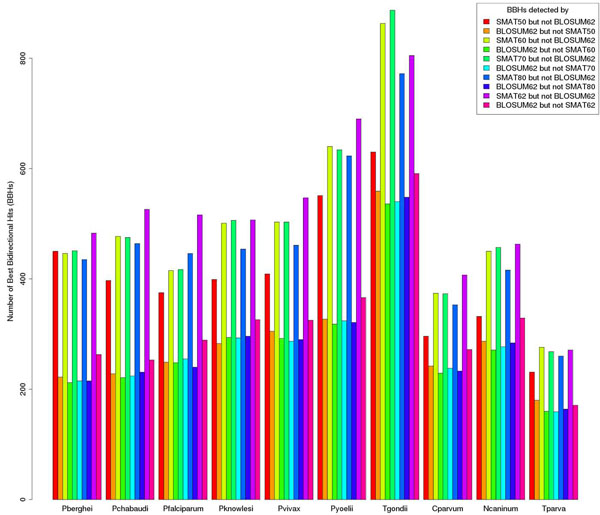
**Number of Best Bidirectional Hits (BBHs) uniquely obtained by SMAT but not by BLOSUM62** The numbers of Best Bidirectional Hits (BBHs) of nine apicomplexan species were calculated against *Arabidopsis thaliana* which are detected by using SMAT50 but not by BLOSUM62 and vice-versa. Similarly BBHs uniquely detected by SMAT60, SMAT70, SMAT80 and SMAT90 matrices but not by BLSOUM62 and vice-versa were calculated. Here for all cases SMAT matrices detect higher number of unique BBHs.

The 'Best Bidirectional Hit' (BBH) is one of the most frequently used methods to determine orthologous pairs. It assumes that a protein pair across two species in which each protein gives back the other protein as being the best hit in the whole other proteome is an orthologous pair [4]. We carried out the BLAST searches for ten apicomplexan species *Plasmodium berghei*, *Plasmodium chabaudi*, *Plasmodium falciparum*, *Plasmodium knowlesi*, *Plasmodium vivax*, *Plasmodium yoelii yoelii*, *Toxoplasma gondii*, *Neospora caninum* and *Cryptosporidium parvum* and *Theileria parva* using BLOSUM (BLOSUM62 & BLOSUM90) and SMAT series of matrices against different eukaryotes and bacteria without an E-value cut-off. A simple shell script was written to calculate the number of Best Bidirectional Hits from these BLAST results for each apicomplexan species against each subject organism. This method generally gives a single BBH for a single protein, although theoretically it may give some many-to-many orthologs [4]. The number of BBHs for all the nine apicomplexan species were calculated from BLAST results against *Arabidopsis thaliana* using BLOSUM62, BLOSUM90, SMAT50, SMAT60, SMAT70, SMAT80 and SMAT90 matrices. In case of *Toxoplasma gondii* (against *Arabidopsis thaliana*), the BLOSUM62 matrix gives BBHs for 2015 proteins, BLOSUM90 for 2191 proteins, SMAT50 for 2239 proteins, SMAT60 for 2337 proteins, SMAT70 for 2325 proteins, SMAT80 for 2317 proteins and SMAT90 for 2339 proteins. Similarly for the other eight apicomplexan species the number of BBHs obtained using matrices of SMAT series is higher compared to that obtained using matrices of BLOSUM series (Additional File 5: Supplementary Figure 3). We also calculated the number of BBHs which are not detected by BLOSUM matrices but by SMAT & vice-versa and found that the number of BBHs obtained by SMAT matrices but not by BLOSUM is higher than that obtained by BLOSUM but not by SMAT matrices (Figure 4 and Additional File 6: Supplementary Figure 6). We further calculated the numbers of BBHs for these apicomplexan species across different eukaryotes using SMAT80, BLOSUM90 and BLOSUM62 matrices. The numbers of BBHs obtained by using SMAT80 matrix were higher than those obtained by using matrices of BLOSUM series (Additional File 7: Supplementary Figure 5 and Additional File 8: Supplementary Figure 6). In fact the numbers of BBHs for these apicomplexan species increase against bacteria as well when using matrices of SMAT series (data not shown). We also estimated the number of apicomplexan proteins which give BBHs against the model eukaryote *Arabidopsis thaliana* with SMAT80 matrix only but not when BLOSUM62 or BLOSUM90 matrix is used. These numbers are 248, 247, 236, 237, 257, 332, 419, 201, 241 and 124 proteins in *P. berghei*, *P. chabaudi*, *P. falciparum*, *P. knowlesi*, *P. vivax*, *P. yoelii*, *Toxoplasma gondii*, *Cryptosporidium parvum*, *Neospora caninum* and *Theileria parva* respectively.

### Examples and applications

#### Best Bidirectional Hit for apicomplexan rhomboid proteases

The proteins PBANKA_110650 (*P. berghei*), PFE0340c (*P. falciparum*) and PVX_097905 (*P. vivax*) do not give any BBH in *Arabidopsis thaliana* using BLOSUM62 and BLOSUM90 matrices. The present annotation (in PlasmoDB version 7.2) of PBANKA_110650 and PFE0340c is rhomboid protease while that of PVX_097905 is a conserved hypothetical protein. Using SMAT80 matrix, we got a single BBH (in *Arabidopsis thaliana*) for all these three proteins and that is ATRBL5 (*Arabidopsis* Rhomboid-like protein-5, gi:15219034). All these four proteins (PBANKA_110650, PFE0340c, PVX_097905 and gi:15219034) have same molecular function (serine-type endopeptidase activity, GO:0004252) and are integral to membrane (GO:0016021). Thereofore we can safely consider that these four proteins are true orthologs of each other and predict that PVX_097905 (presently labelled as conserved hypothetical protein in PlasmoDB version 7.2) is a rhomboid protease.

#### Best Bidirectional Hit for splicing factor subunit of *Cryptosporidium parvum*

The cgd2_1480 is a large subunit of U2 snRNP auxiliary factor of *Cryptosporidium parvum* which do not give any BBH in *Arabidopsis thaliana* using BLOSUM62 and BLOSUM90 matrices. The SMAT80 matrix gives BBH for this protein in *Arabidopsis thaliana* (gid: 30696485) with same annotation. The E-values are 2e-10 and 5e-12 when *Cryptosporidium parvum* is used as query and subject respectively.

#### Alignment of experimentally characterized glutathione S-transferase from an apicomplexan

*P. falciparum* GST (glutathione S-transferase, PF14_0187) is an experimentally characterized protein (Molecular Function GO:0004364, glutathione transferase activity, evidence code IDA, source:http://www.plasmodb.org) [6,7] which has Best Bidirectional Hits in all other five plasmodia studied in this paper. *P. falciparum* GST (Pf-GST) and its orthologs in other plasmodia show very low level of conservation with yeast GST (gi: 6322968) using matrices of BLOSUM series. We observed significant improvement in pair-wise alignment of this experimentally characterized Pf-GST with yeast GST using PfFSmat60 matrix by fasta program (Additional File 9: Supplementary Figure 7) as well as by water program. This significant improvement in alignment by PfFSmat60 matrix was also observed for other apicomplexan GSTs with yeast GST indicating the usefulness of these matrices for improving the alignment of diverged apicomplexan proteins moreover this improvement was observed with both fasta and water programs (Table 1).

**Table 1:** Pairwise alignments of plasmodia GSTs with yeast GST.

EMBOSS/water program	BLOSUM62 matrix		PfFSmat60 matrix	
Organism	Length	Similarity (%)	Length	Similarity (%)
*P. berghei* (PBANKA_102390) *P. chabaudi* (PCHAS_102470) *P. falciparum* (PF14_0187) *P. knowlesi* (PKH_132970) *P. vivax* (PVX_085515) *P. yoelii* (PY05088)	200 208 206 200 200 208	39.50 36.50 37.40 38.50 38.00 36.50	247 247 248 246 250 250	55.90 56.30 57.30 57.70 57.60 54.80
				
FASTA program	**BLOSUM50 matrix**		**PfFSmat60 matrix**	
Organism	Length	E-value	Length	E-value
*P. berghei* (PBANKA_102390) *P. chabaudi* (PCHAS_102470) *P. falciparum* (PF14_0187) *P. knowlesi* (PKH_132970) *P. vivax* (PVX_085515) *P. yoelii* (PY05088)	71 22 22 190 166 73	0.05 0.15 1 0.05 0.07 0.10	229 229 233 231 231 231	3.90E-041 5.00E-039 1.80E-016 2.30E-042 1.00E-042 2.90E-037

#### Alignment of experimentally characterized protein kinase

The eukaryotic protein kinases (ePKs) are a large family of enzymes with crucial roles in most cellular processes; hence malarial ePKS represent potential drug targets [8]. In case of *Plasmodium falciparum*, PF11_0220 (Molecular Function GO:0004672, protein kinase activity, evidence code IDA, source:http://www.plasmodb.org) is a known protein kinase which shows poor alignment with known yeast protein kinase with BLOSUM series of matrices. The pairwise alignment of PF11_0220 against PIK-related protein kinase and rapamycin target of *Saccharomyces cerevisiae* (gi: 6322526) was performed with standard and PfFSmat60 matrices by fasta program (FASTA package, version 3) [9] (Additional File 10: Supplementary Figure 8) and water program (EMBOSS package, version 6.3.1) (data not shown). We observed improvement in alignment by using PfFSmat60 over other matrices irrespective of program used for alignment. With fasta program, BLOSUM50 gave an alignment score of 23.4 bits at an E-value 0.31 with an overlap of only 71 amino acid residues, BLOSUM100 gave an alignment score of 18.1 bits at an E-value 1 with an overlap of only 16 amino acids. PfFSmat60 gave an alignment score of 4872.5 bits at an E-value 0.0 and the overlap was 1990 amino acid residues. PAM2, a similar entropy matrix, gave an insignificant alignment with a score of 22.8 bits and an *E*-value of 0.43 for an overlap of only 6 amino acids.

#### Alignment of two experimentally known Acyl CoA binding proteins

Acyl CoA Binding Proteins (ACBPs) are generally small (10 kD) highly conserved proteins found in all four eukaryotic kingdoms Animalia, Plantae, Fungi, Protista and only eleven eubacterial species but not in any other known bacterial species or in archaea till now [10]. The long type ACBPs containing ankyrin repeats have been characterized experimentally in *Cryptosporidium parvum* and *Arabidopsis thaliana*, Zeng *et.al *[11] characterized the CpACBP1 (*Cryptosporidium parvum* Acyl CoA binding protein) containing acyl CoA binding domain and ankyrin repeats while Xiao *et. al *[12,13] studied similar type of ACBP with ankyrin repeats from *Arabidopsis thaliana* ACBP1. Therefore we can safely consider *Arabidopsis* ACBP1 to be a true ortholog of CpACBP1. We performed pairwise alignment of these two ACBPs using different matrices by fasta and water programs. We observed significant improvement in alignment by PfFSmat60 though not much statistically but it was expected as these proteins (ACBPs) are highly conserved and show good alignment even with standard matrices. Our purpose here was to see alignment of an apicomplexan protein with its experimentally known ortholog using standard and PfFSmat60 matrices (Additional File 11: Supplementary Table 5).

#### Bi-functional enzyme of shikimate pathway across apicomplexan genomes

**Figure 5:**
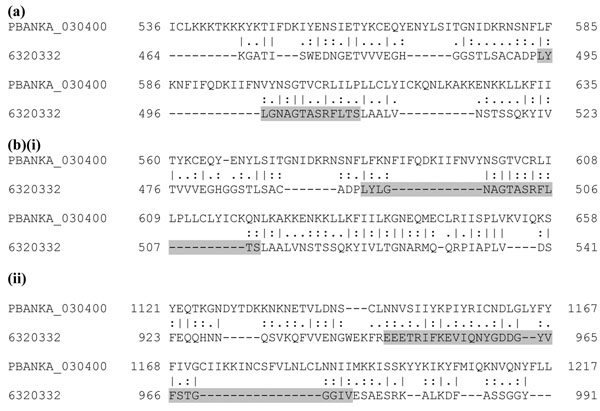
**Alignment extension with PfFSmat60 matrix for *P. berghei* probable bi-functional enzyme of shikimate pathway.** The sequences compared here are the *P. bergheii* probable bi-functional enzyme having EPSP and SK activities, PBANKA_030400 and well characterized yeast multifunctional protein, Aro1p (gi:6320332). (**a**) The alignment with BLOSUM50 showing the aligned motif regions for only EPSP synthase I motif (gray shading). (**b**) The alignments extended by PfFSmat60 for both the EPSP synthase I and shikimate kinase motifs are represented as (i) and (ii) respectively. The water program (EMBOSS package, version 6.3.1) was used for alignment.

The shikimate pathway plays an important role in the survival of Apicomplexans. The enzymatic activities of six out of seven shikimate pathway enzymes have been detected in crude extracts of either *P. falciparum* or *Toxoplasma gondii* or both [14,15]. The chorismate synthase is the only enzyme that has been identified in Apicomplexa to date. Moreover the absence of this pathway in mammals makes it a probable drug target for apicomplexan parasites [16]. We have already reported the better alignment of a probable *P. falciparum* bi-functional protein, PFB0280w having EPSP (5-enolpyruvylshikimate-3-phosphate) and SK (shikimate kinase) domains with the yeast AROM complex (gi: 6320332) [2]. We have performed the alignments for orthologs of PFB0280w in *P. berghei* (PBANKA_030400), *P. chabaudi* (PCHAS_030620), *P. knowlesi* (PKH_041350), *P. vivax* (PVX_003750) and *P. yoelii* (PY00069) with yeast AROM complex and observed that the aligned regions had both the SK and EPSP domains with PfFSmat60 matrix while the standard matrix had only the SK domains aligned. An example alignment of *P. berghei* (PBANKA_030400) and yeast AROM complex is provided in Figure 5, while the remaining alignments have been provided in Additional Files 12, 13, 14 and 15: Supplementary Figures 9, 10, 11 and 12. The length of overlap and the percentage similarity obtained for alignment of yeast AROM complex with this protein across all plasmodia proteins with PfFSmat60 and BLOSUM matrices is provided in Additional File 16: Supplementary Table 4.

#### Example of a missing metabolic enzyme - Acylglycerol lipase

Recently Mohanty and Srinivasan [17] have attempted to identify some of the missing enzymes from the parasite genome using multiple profiles for every protein domain family. One of the predicted missing enzymes was a conserved *Plasmodium* protein with an unknown function, MAL7P1.156. They predicted it to be acylglycerol lipase associated with glycerol biosynthesis pathway. *Saccharomyces cerevisiae* has four experimentally characterized triglyceride lipases tgl2p (gi:6320263), tgl3p (gi:6323973), tgl4p (gi:6322942) and tgl5p (gi:6324655). We submitted MAL7P1.156 as query and compared it with all these four yeast lipases and the best match was observed with tgl5p (gi:6324655) (BLOSUM50 E-value 0.15; BLOSUM100 E-value 1.0; PfFSmat60 E-value 8.4e-156 and PAM2 E-value 1.0) (Additional File 17: Supplementary Table 3). PfFSmat60 gives much larger and qualitatively better alignment compared to standard matrices as it covers the functionally important whole patatin domain (183-388 residues) of yeast lipase tgl5p (gi:6324655) (Additional File 18: Supplementary Figure 13).

## Conclusions

The PfSSM (*Plasmodium falciparum* Specific Substitution Matrices) were developed basically for *P. falciparum* and particularly for those proteins which do not find their orthologs in other eukaryotes or show very poor alignment with their orthologs. In this study, database searches, best bidirectional hits and the improved pairwise alignment of apicomplexan proteins have shown that these matrices perform better for apicomplexan species other than *Plasmodium falciparum* and they can be thus helpful in improving the annotation of the same. To provide the access to these matrices for researchers working on apicomplexan species, we developed a web server ApicoAlign for detecting orthologs and aligning apicomplexan proteins. The real importance of this tool will be for those apicomplexan proteins which do not give any ortholog in other eukaryotes or show poor alignment at sequence level using matrices of BLOSUM and PAM series.

## Methods

### Amino acid composition of different apicomplexan species

We compared the amino acid composition for all the proteins of ten apicomplexan genomes (*Plasmodium berghei*, *Plasmodium chabaudi*, *Plasmodium falciparum*, *Plasmodium knowlesi*, *Plasmodium vivax*, *Plasmodium yoelii yoelii*, *Toxoplasma gondii*, *Cryptosporidium parvum*, *Neospora caninum* and *Theileria parva*) with that of non-apicomplexan *Mycobacterium tuberculosis* genome. The proteins having the terms “hypothetical”, “putative” and “unknown function” were removed in all the genomes. A matrix (20 columns for 20 amino acids and where each row represents a protein) was generated by calculating the fraction of each amino acid in each protein. The mean was calculated for each column (amino acid) and thus for each genome we got 20 means for 20 amino acids. The amino acids were divided in four categories based on their properties namely non-polar amino acids (glycine, alanine, valine, leucine, isoleucine, methionine, proline, phenylalanine & tryptophan), polar amino acids with no charge (serine, tyrosine, threonine, cysteine, asparagine & glutamine), positively charged amino acids (arginine, histidine & lysine) and negatively charged amino acids (aspartate & glutamate). Next the means of amino acids of each category were used to calculate the p-value of student t-test between any two genomes. Next, the P-values for a two tailed t-test for correlated samples were calculated for each individual amino acid fraction obtained from the ortholog set of these organisms. The higher the p-value of t-test, the closer will be the two genomes in terms of amino acid composition.

### Datasets used

The complete protein datasets of *Plasmodium berghei*, *Plasmodium chabaudi*, *Plasmodium falciparum*, *Plasmodium knowlesi*, *Plasmodium vivax* and *Plasmodium yoelii yoelii* were downloaded from PlasmoDB release 7.0 [7], that of *Toxoplasma gondii* and *Neospora caninum* were downloaded from ToxoDB release 6.2 [18], that of *Cryptosporidium parvum* from CryptoDB release 4.3 [19] and for rest other organisms used in this study and for the web server the whole protein datasets were downloaded from NCBI ftp site.

### Softwares/programs used

The examples of pairwise alignment in this paper use fasta program (FASTA package, version 3) and water program (EMBOSS package, version 6.3.1) for comparison while pairwise alignment section of ApicoAlign uses water program only. All the BLAST searches have been performed using the standalone version of BLASTp program [21] obtained from NCBI by anonymous ftp (ftp://ftp.ncbi.nih.gov/toolbox/ncbi_tools/old/20051206). The BLAST source code was modified to accept the Smat series of matrices. The web page of ApicoAlign uses HTML, CSS and JavaScript while the background programs have been written in Perl/CGI. Awk, sed and perl have been used as shell scripts for finding best non-self hits common to two matrices, Best Bidirectional Hits between two organisms and for other small purposes. R package (R-2.9.1 version, http://www.r-project.org/) was used for making graphs. VassarStats, a website for statistical computation (http://faculty.vassar.edu/lowry/VassarStats.html) was used for calculating the two tailed P-values for correlated samples of amino acid fractions.

## Availability and requirements

**Project name:** ApicoAlign

**Project home page:**http://www.cdfd.org.in/apicoalign/

**Operating system(s):** Platform independent and it is not web browser specific.

**Programming language:** Perl/CGI, HTML/CSS and JavaScript.

**Other requirements:** only internet and any web browser like firefox or internet explorer.

**Any restrictions to use by non-academics:** No restriction.

## Authors' contributions

JA has developed the ApicoAlign Perl-CGI web application, extended the use of matrices for other apicomplexan species and wrote the manuscript, UP developed PfSSM (*Plasmodium falciparum* Specific Substitution Matrices), showed that they perform better for *P. falciparum* and helped in the compilation of manuscript and designing ApicoAlign. Akash Ranjan co-ordinated and owned the study. All authors read and approved the final manuscript.

## Competing interests

The authors declare that they have no competing interests.

## Supplementary Material

Additional file 1 **Supplementary Table S1: Similarities in amino acid composition for different apicomplexan species** T-test P-values (two-tailed ) for the amino acid frequency of all 20 amino acids across proteins of *P. falciparum* vs. *Mycobacterium tuberculosis* (control) and other Apicomplexans orthologs. Click here for file

Additional file 2 **Supplementary Table S2: Performance of SMAT80 over BLOSUM90 for different apicomplexan species against non-redundant database** The E-values and bits scores of non-self best hits commmon to SMAT80 and BLOSUM90 were compared for *P. berghei* (sheet1), *P. chabaudi* (sheet2), *P. knowlesi* (sheet3), *P. vivax* (sheet4), *P. yoelii yoelii* (sheet5), *Toxoplasma gondii* (sheet6), *Cryptosporidium parvum* (sheet7), *Neospora caninum* (sheet8) and *Theileria parva* (sheet9). Click here for file

Additional file 3 **Supplementary Figure 1: Comparison of E-values & bit scores given by SMAT80 and BLOSUM62 matrices** BLAST searches were performed against non-redundant (nr) database for nine Apicomplexan species (the labels on X-axis: Pberghei for *Plasmodium berghei*, Pchabaudi for *Plasmodium chabaudi*, Pknowlesi for *Plasmodium knowlesi*, Pvivax for *Plasmodium vivax*, Pyoelii for *Plasmodium yoelii yoelii*, Tgondii for *Toxoplasma gondii*, Cparvum for *Cryptosporidium parvum*, Ncaninum for *Neospora caninum* and Tparva for *Theileria parva*) using SMAT80 and BLOSUM62 matrix. The best non-self hits common to both matrices from these BLAST results were divided in eight categories shown in the legend at topleft position of figure. The percentage for each category was calculated and it was observed that most of the apicomplexan proteins fall in first two categories that means most of apicomplexan proteins give better or similar E-values and better bit scores with SMAT80 compared to BLOSUM62 matrix. Click here for file

Additional file 4 **Supplementary Figure 2 Comparison of percent identity, alignment length and mismatches given by SMAT80 and BLOSUM62 matrices** BLAST searches were performed against non-redundant (nr) database for nine Apicomplexan species (the labels on X-axis: Pberghei for *Plasmodium berghei*, Pchabaudi for *Plasmodium chabaudi*, Pknowlesi for *Plasmodium knowlesi*, Pvivax for *Plasmodium vivax*, Pyoelii for *Plasmodium yoelii yoelii*, Tgondii for *Toxoplasma gondii*, Cparvum for *Cryptosporidium parvum*, Ncaninum for *Neospora caninum* and Tparva for *Theileria parva*) using SMAT80 and BLOSUM62 matrix. The best non-self hits common to both matrices were filtered out from these BLAST results. The percent identity, alignment length and number of mismatches were divided in two categories- better or poor using SMAT80 compared to BLOSUM62 and the numbers of proteins for these categories were calculated. We see here a more number of proteins belonging to better category in each case. Click here for file

Additional file 5 **Supplementary Figure 3: Comparison of SMAT with BLOSUM series in terms of Best Bidirectional Hits (BBH)** The Best Bidirectional Hits (BBHs) were extracted from BLAST results of nine apicomplexan species studied here against *Arabidopsis thaliana* using BLOSUM62, BLOSUM90, SMAT50, SMAT60, SMAT70, SMAT80 and SMAT90 matrices. The colour of the bar corresponds to the matrix in the legend at topleft position of figure using which BBHs were calculated. Click here for file

Additional file 6 **Supplementary Figure 4: Number of Best Bidirectional Hits (BBHs) uniquely obtained by SMAT but not by BLOSUM90** The numbers of Best Bidirectional Hits (BBHs) of nine apicomplexan species were calculated against *Arabidopsis thaliana* which are detected by using SMAT50 but not by BLOSUM90 and vice-versa. Similarly BBHs uniquely detected by SMAT60, SMAT70, SMAT80 and SMAT90 matrices but not by BLSOUM90 and vice-versa were calculated. In *P. vivax*, *T. gondii*, *C. parvum*, *N. caninum* and *T. parva* BLOSUM90 gives slightly higher number of unique BBHs compared to SMAT50 but for rest other cases SMAT matrices generally pick higher number of unique BBHs. Click here for file

Additional file 7 **Supplementary Figure 5: Best Bidirectional Hits (BBH) for proteins of different plasmodia using SMAT80 and BLOSUM matrices** The Best Bidirectional Hits (BBHs) were extracted from BLAST results of six *Plasmodium* species: *Plasmodium berghei* (Pb), *Plasmodium chabaudi* (Pc), *Plasmodium falciparum* (Pf), *Plasmodium knowlesi* (Pk), *Plasmodium vivax* (Pv) and *Plasmodium yoelii yoelii* (Py) using SMAT80, BLOSUM90 and BLOSUM62 matrices. The labels on x-axis are two letter abbreviation of organism followed by name of the matrix used like Pb_SMAT80 means number of BBHs for *Plasmodium berghei* using SMAT80 matrix. The colour of the bar corresponds to the organism in the legend at top right position of figure against which BBHs were calculated. Click here for file

Additional file 8 **Supplementary Figure 6: Best Bidirectional Hits (BBH) for apicomplexan proteins other than plasmodia using SMAT80 and BLOSUM matrices** The Best Bidirectional Hits (BBHs) were extracted from BLAST results of four apicomplexan species: *Toxoplasma gondii* (Tg), *Cryptosporidium parvum* (Cp), *Neospora caninum* (Nc) and *Theileria parva* (Tp) using SMAT80, BLOSUM90 and BLOSUM62 matrices. The labels on x-axis are two letter abbreviation of organism followed by name of the matrix used like Tg_SMAT80 means number of BBHs for *Toxoplasma gondii* using SMAT80 matrix. The colour of the bar corresponds to the organism in the legend at top right position of figure against which BBHs were calculated. Click here for file

Additional file 9 **Supplementary Figure 7: Alignment extension of experimentally characterized *P. falciparum* GST with PfFSmat60 matrix** The sequences compared here are the experimentally characterized Glutathione S-transferase (GST) of *P. falciparum*, PF14_0187 and yeast Gtt2p (Glutathione S-transferase capable of homodimerization, gi:6322968). (a) The alignment with BLOSUM50 was only 22 amino acids (59 to 79 for query and 72 to 93 for subject). (b) A significantly improved alignment of 233 (1 to 209 for query and 15 to 232 for subject) amino acids is achieved using PfFSmat60 matrix. The fasta program (FASTA package, version 3) was used for alignment. Click here for file

Additional file 10 **Supplementary Figure 8: Alignment extension of experimentally characterized *P. falciparum* kinase with PfFSmat60 matrix** The sequences compared are the experimentally characterized *P. falciparum* protein kinase (PF11_0220) and PIK-related protein kinase and rapamycin target of *Saccharomyces cerevisiae* (gi: 6322526). While BLOSUM50 gave an alignment score of 23.4 bits at an E-value 0.31 with an overlap of only 71 amino acid residues (637-707:2090-2157), PfFSmat60 gave an alignment score of 4872.5 bits at an E-value 0.0 and the overlap was 1990 amino acid residues (1-1675:375-2307). The fasta program (FASTA package, version 3) was used for alignment. Click here for file

Additional file 11 **Supplementary Table S5: Pairwise alignments of *Cyptosporidium parvum* ACBP1 (cgd1_1140) against *Arabidopsis thaliana* ACBP1 (gi:15238757)** The pairwise alignment was performed between two experimentally characterized proteins *Cyptosporidium parvum* ACBP1 (cgd1_1140) and *Arabidopsis thaliana* ACBP1 (gi:15238757) using water program (EMBOSS package) and FASTA program. Click here for file

Additional file 12 **Supplementary Figure 9: Alignment extension of probable *P. chabaudi* bi-functional enzyme of the shikimate pathway** The sequences compared here are the *P. chabaudi* hypothetical protein, PCHAS_041350 and yeast multifunctional protein, Aro1p (gi:6320332). (a) The alignment with BLOSUM50 showing the aligned motif regions for only EPSP synthase I motif (gray shading). (b) The alignment extended by PfFSmat60 for both the EPSP synthase I and shikimate kinase motifs represented as (i) and (ii) respectively. The fasta program (FASTA package, version 3) was used for alignment. Click here for file

Additional file 13 **Supplementary Figure 10: Alignment extension of probable *P. knowlesi* bi-functional enzyme of the shikimate pathway** The sequences compared here are the *P. knowlesi* hypothetical protein, PKH_041350 and yeast multifunctional protein, Aro1p (gi:6320332). (a) The alignment with BLOSUM50 showing the aligned motif regions for only EPSP synthase I motif (gray shading). (b) The alignment extended by PfFSmat60 for both the EPSP synthase I and shikimate kinase motifs represented as (i) and (ii) respectively. The fasta program (FASTA package, version 3) was used for alignment. Click here for file

Additional file 14 **Supplementary Figure 11: Alignment extension of probable *P. vivax* bi-functional enzyme of the shikimate pathway** The sequences compared here are the *P. vivax* hypothetical protein, PVX_003750 and yeast multifunctional protein, Aro1p (gi:6320332). (a) The alignment with BLOSUM50 showing the aligned motif regions for only EPSP synthase I motif (gray shading). (b) The alignment extended by PfFSmat60 for both the EPSP synthase I and shikimate kinase motifs represented as (i) and (ii) respectively. The fasta program (FASTA package, version 3) was used for alignment. Click here for file

Additional file 15 **Supplementary Figure 12: Alignment extension of probable *P. yoelii* bi-functional enzyme of the shikimate pathway** The sequences compared here are the *P. yoelii* hypothetical protein, PY00069 and yeast multifunctional protein, Aro1p (gi:6320332). (a) The alignment with BLOSUM50 showing the aligned motif regions for only EPSP synthase I motif (gray shading). (b) The alignment extended by PfFSmat60 for both the EPSP synthase I and shikimate kinase motifs represented as (i) and (ii) respectively. The fasta program (FASTA package, version 3) was used for alignment. Click here for file

Additional file 16 **Supplementary Table S4: Pairwise alignments of probable plasmodia bifunctional proteins of shikimate pathway with yeast AROM complex** The pairwise alignments of probable plasmodia bifunctional proteins of shikimate pathway with yeast AROM complex using water program (EMBOSS package) and FASTA program. Click here for file

Additional file 17 **Supplementary Table S3: Pairwise alignments of MAL7P1.156 against four known yeast triglyceride lipases** The table shows the E-values, bits scores and alignment length of pairwise alignments between probable *P. falciparum* acyl glycerol lipase (MAL7P1.156) and four known yeast triglyceride lipases (tgl2p, tgl3p, tgl4p and tgl5p). Click here for file

Additional file 18 **Supplementary Figure 13: Alignment of probable missing enzyme of *P. falciparum* glycerol biosynthesis pathway** The sequences compared are conserved protein in *Plasmodium* sps. with unknown function, MAL7P1.156 and yeast triacylglycerol lipase tgl5p which has patatin domain for lipase activity spanning 183 to 388 residues. (a) The alignment with BLOSUM50 covered only few residues of patatin domain (grey shaded). (b) The alignment generated with PfFSmat60 covered whole patatin domain of subject sequence. The fasta program (FASTA package, version 3) was used for alignment. Click here for file
