# Supplementary figures and images for: ApicoAlign: an alignment and sequence search tool for apicomplexan proteins
Source: BMC Genomics. 2011 Nov 30;12(Suppl 3):S6. doi: 10.1186/1471-2164-12-S3-S6 (PMC3333189; doi:10.1186/1471-2164-12-S3-S6)

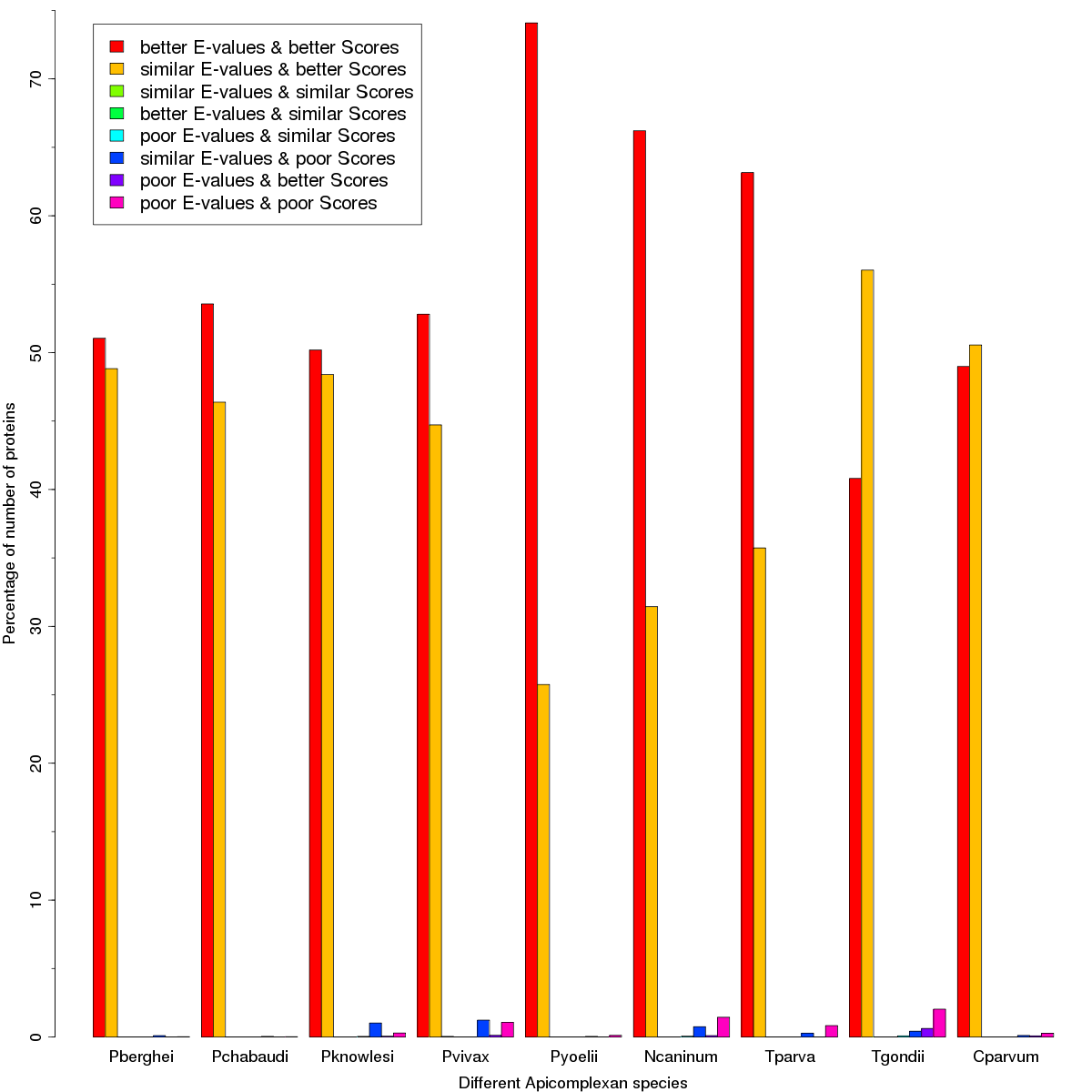

Supplement: Additional file 3 — Supplementary Figure 1: Comparison of E-values & bit scores given by SMAT80 and BLOSUM62 matrices BLAST searches were performed against non-redundant (nr) database for nine Apicomplexan species (the labels on X-axis: Pberghei for Plasmodium berghei, Pchabaudi for Plasmodium chabaudi, Pknowlesi for Plasmodium knowlesi, Pvivax for Plasmodium vivax, Pyoelii for Plasmodium yoelii yoelii, Tgondii for Toxoplasma gondii, Cparvum for Cryptosporidium parvum, Ncaninum for Neospora caninum and Tparva for Theileria parva) using SMAT80 and BLOSUM62 matrix. The best non-self hits common to both matrices from these BLAST results were divided in eight categories shown in the legend at topleft position of figure. The percentage for each category was calculated and it was observed that most of the apicomplexan proteins fall in first two categories that means most of apicomplexan proteins give better or similar E-values and better bit scores with SMAT80 compared to BLOSUM62 matrix. [file 1471-2164-12-S3-S6-S3.png]

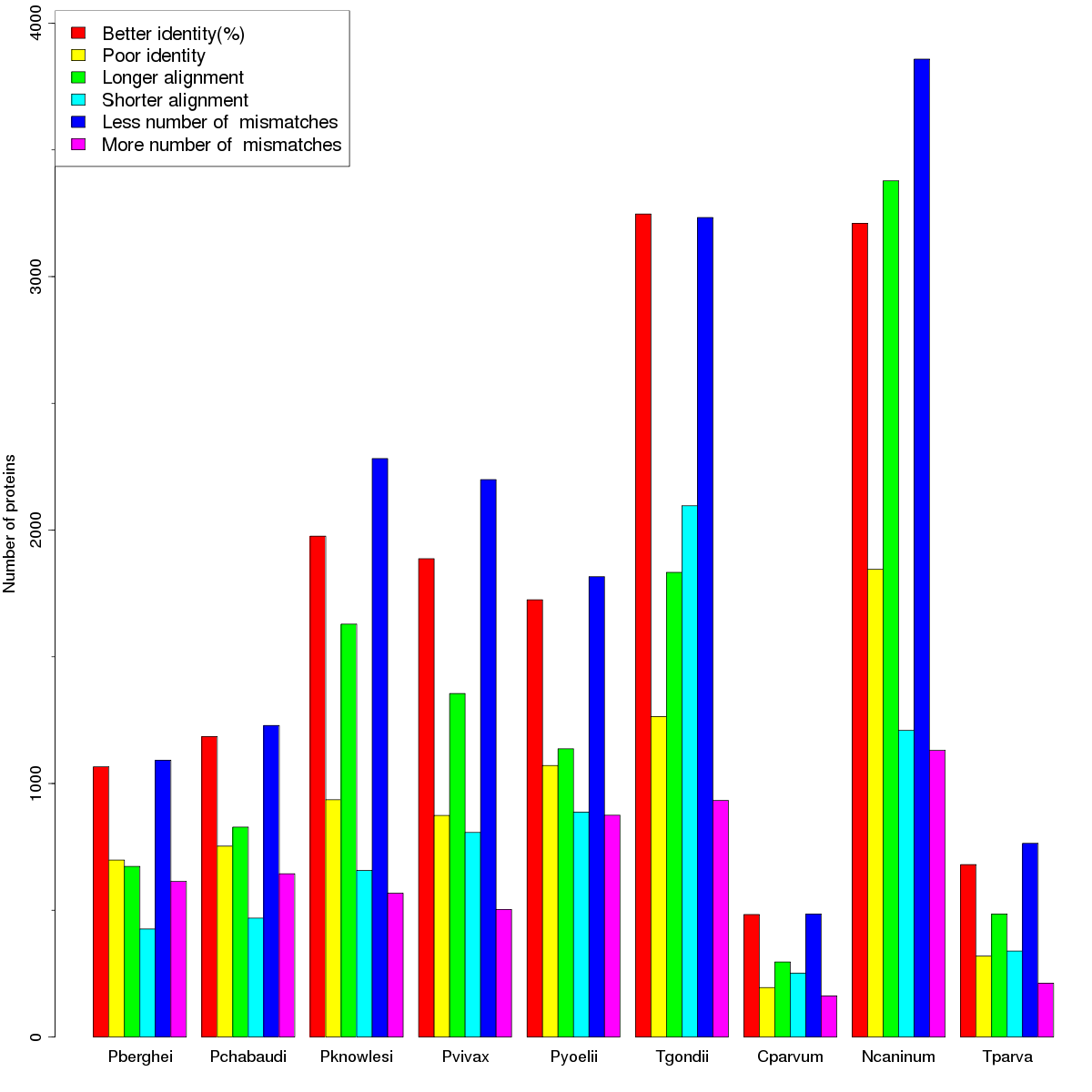

Supplement: Additional file 4 — Supplementary Figure 2 Comparison of percent identity, alignment length and mismatches given by SMAT80 and BLOSUM62 matrices BLAST searches were performed against non-redundant (nr) database for nine Apicomplexan species (the labels on X-axis: Pberghei for Plasmodium berghei, Pchabaudi for Plasmodium chabaudi, Pknowlesi for Plasmodium knowlesi, Pvivax for Plasmodium vivax, Pyoelii for Plasmodium yoelii yoelii, Tgondii for Toxoplasma gondii, Cparvum for Cryptosporidium parvum, Ncaninum for Neospora caninum and Tparva for Theileria parva) using SMAT80 and BLOSUM62 matrix. The best non-self hits common to both matrices were filtered out from these BLAST results. The percent identity, alignment length and number of mismatches were divided in two categories- better or poor using SMAT80 compared to BLOSUM62 and the numbers of proteins for these categories were calculated. We see here a more number of proteins belonging to better category in each case. [file 1471-2164-12-S3-S6-S4.png]

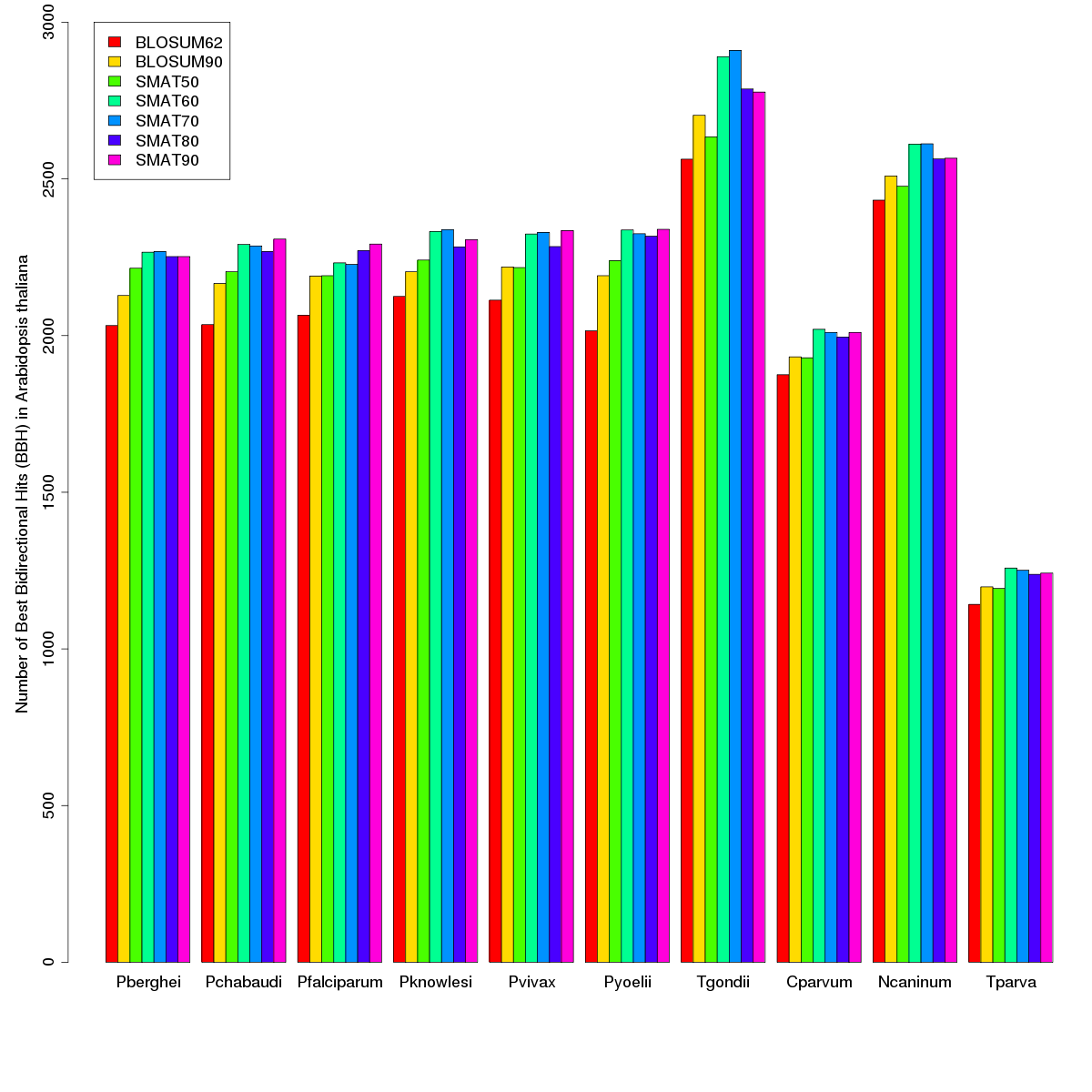

Supplement: Additional file 5 — Supplementary Figure 3: Comparison of SMAT with BLOSUM series in terms of Best Bidirectional Hits (BBH) The Best Bidirectional Hits (BBHs) were extracted from BLAST results of nine apicomplexan species studied here against Arabidopsis thaliana using BLOSUM62, BLOSUM90, SMAT50, SMAT60, SMAT70, SMAT80 and SMAT90 matrices. The colour of the bar corresponds to the matrix in the legend at topleft position of figure using which BBHs were calculated. [file 1471-2164-12-S3-S6-S5.png]

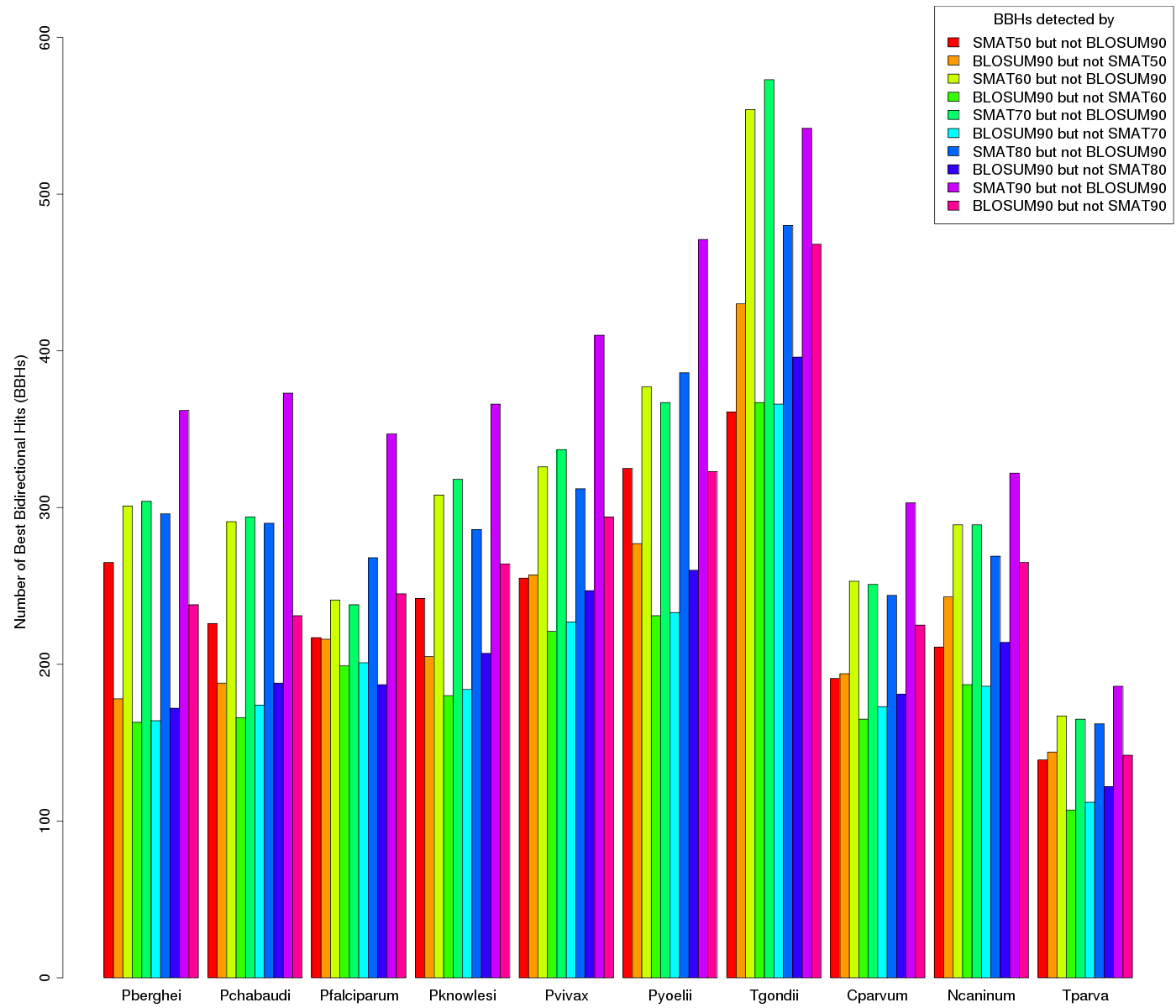

Supplement: Additional file 6 — Supplementary Figure 4: Number of Best Bidirectional Hits (BBHs) uniquely obtained by SMAT but not by BLOSUM90 The numbers of Best Bidirectional Hits (BBHs) of nine apicomplexan species were calculated against Arabidopsis thaliana which are detected by using SMAT50 but not by BLOSUM90 and vice-versa. Similarly BBHs uniquely detected by SMAT60, SMAT70, SMAT80 and SMAT90 matrices but not by BLSOUM90 and vice-versa were calculated. In P. vivax, T. gondii, C. parvum, N. caninum and T. parva BLOSUM90 gives slightly higher number of unique BBHs compared to SMAT50 but for rest other cases SMAT matrices generally pick higher number of unique BBHs. [file 1471-2164-12-S3-S6-S6.png]

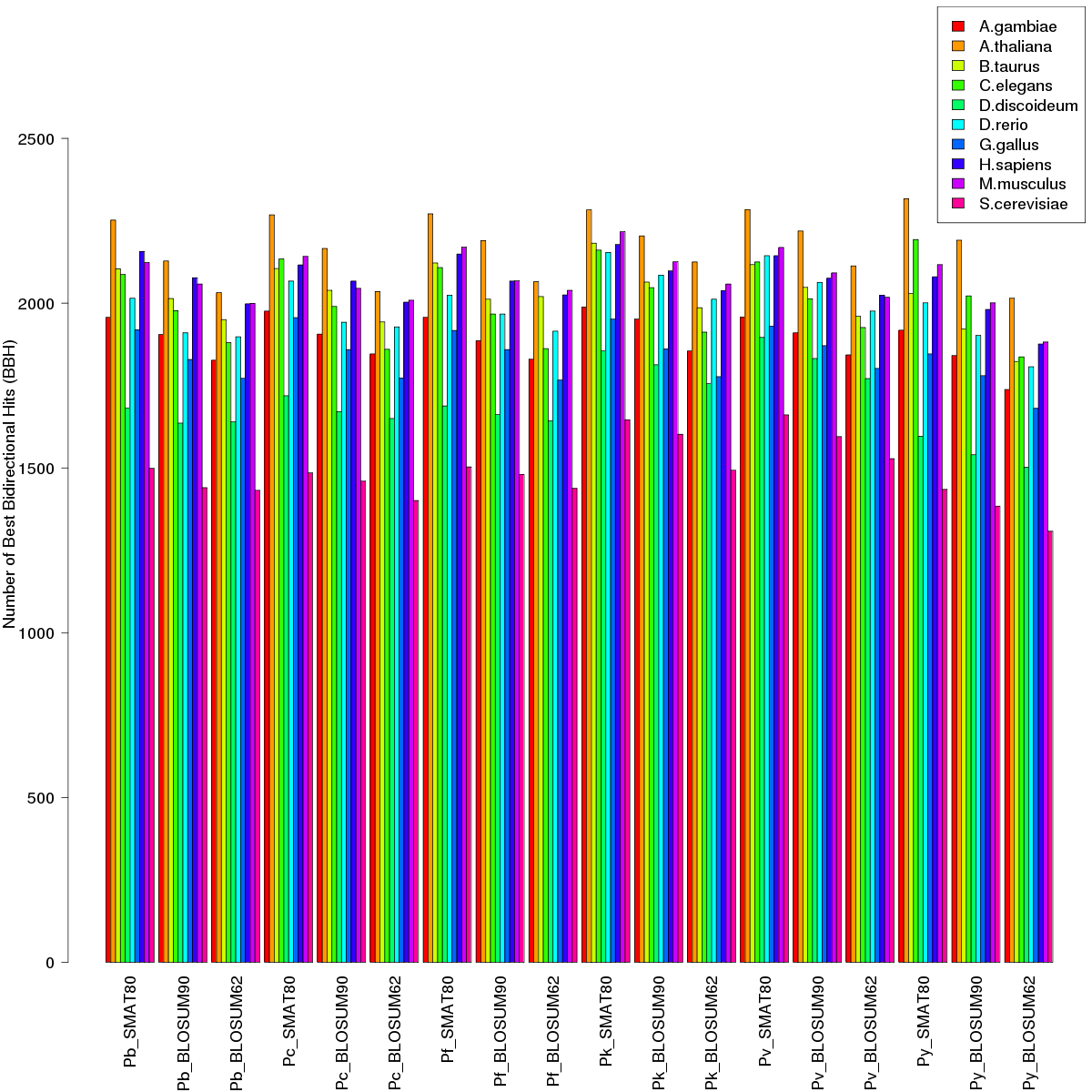

Supplement: Additional file 7 — Supplementary Figure 5: Best Bidirectional Hits (BBH) for proteins of different plasmodia using SMAT80 and BLOSUM matrices The Best Bidirectional Hits (BBHs) were extracted from BLAST results of six Plasmodium species: Plasmodium berghei (Pb), Plasmodium chabaudi (Pc), Plasmodium falciparum (Pf), Plasmodium knowlesi (Pk), Plasmodium vivax (Pv) and Plasmodium yoelii yoelii (Py) using SMAT80, BLOSUM90 and BLOSUM62 matrices. The labels on x-axis are two letter abbreviation of organism followed by name of the matrix used like Pb_SMAT80 means number of BBHs for Plasmodium berghei using SMAT80 matrix. The colour of the bar corresponds to the organism in the legend at top right position of figure against which BBHs were calculated. [file 1471-2164-12-S3-S6-S7.png]

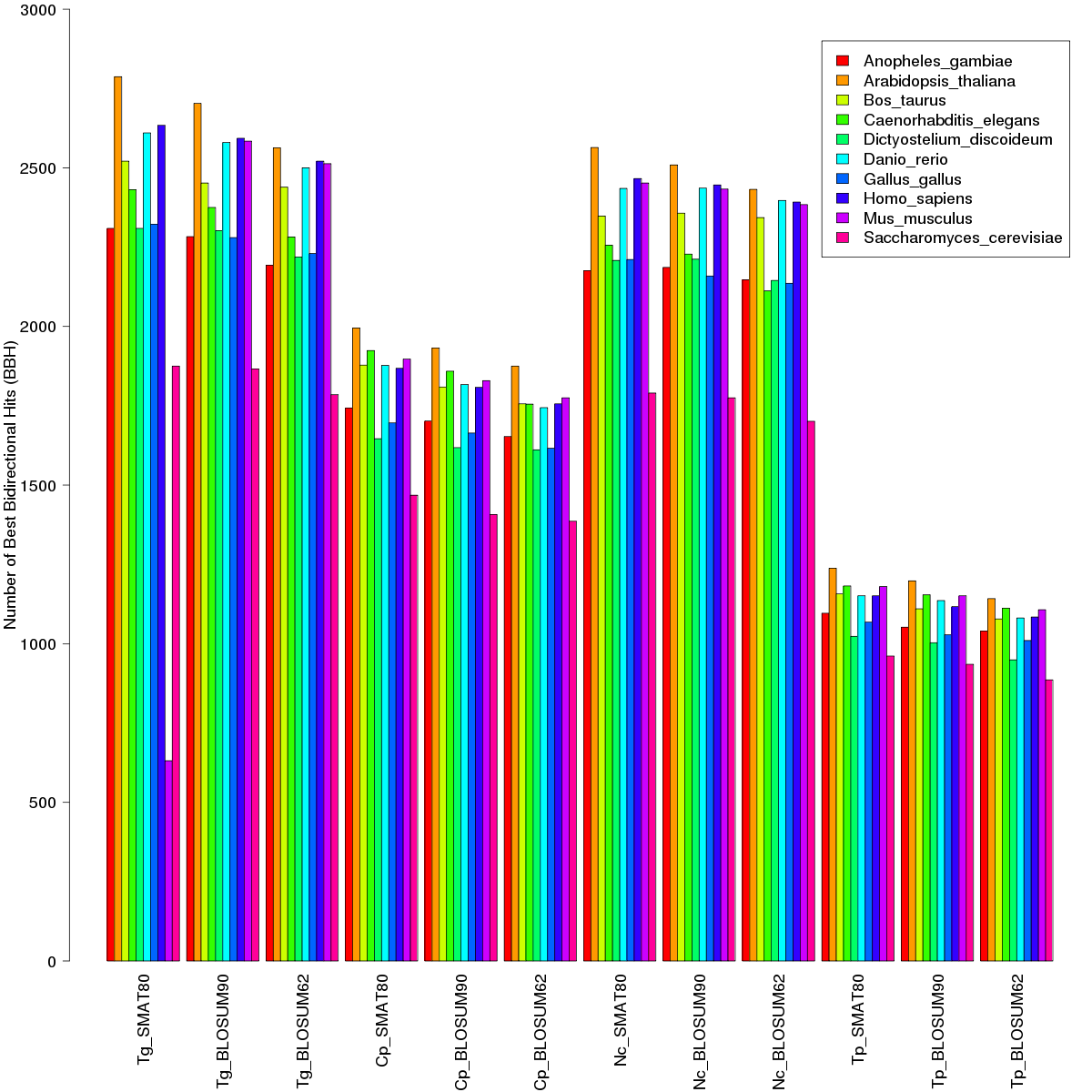

Supplement: Additional file 8 — Supplementary Figure 6: Best Bidirectional Hits (BBH) for apicomplexan proteins other than plasmodia using SMAT80 and BLOSUM matrices The Best Bidirectional Hits (BBHs) were extracted from BLAST results of four apicomplexan species: Toxoplasma gondii (Tg), Cryptosporidium parvum (Cp), Neospora caninum (Nc) and Theileria parva (Tp) using SMAT80, BLOSUM90 and BLOSUM62 matrices. The labels on x-axis are two letter abbreviation of organism followed by name of the matrix used like Tg_SMAT80 means number of BBHs for Toxoplasma gondii using SMAT80 matrix. The colour of the bar corresponds to the organism in the legend at top right position of figure against which BBHs were calculated. [file 1471-2164-12-S3-S6-S8.png]
