# Supplementary material for: ApicoAlign: an alignment and sequence search tool for apicomplexan proteins
Source: BMC Genomics. 2011 Nov 30;12(Suppl 3):S6. doi: 10.1186/1471-2164-12-S3-S6 (PMC3333189; doi:10.1186/1471-2164-12-S3-S6)
Supplement: Additional file 9 — Supplementary Figure 7: Alignment extension of experimentally characterized P. falciparum GST with PfFSmat60 matrix The sequences compared here are the experimentally characterized Glutathione S-transferase (GST) of P. falciparum, PF14_0187 and yeast Gtt2p (Glutathione S-transferase capable of homodimerization, gi:6322968). (a) The alignment with BLOSUM50 was only 22 amino acids (59 to 79 for query and 72 to 93 for subject). (b) A significantly improved alignment of 233 (1 to 209 for query and 15 to 232 for subject) amino acids is achieved using PfFSmat60 matrix. The fasta program (FASTA package, version 3) was used for alignment. [file 1471-2164-12-S3-S6-S9.doc]

**(a)**
 30 40 50 60 70 80
PF14_0 EYTDKRFGVNGDAFVEFKNFKKEKDTPFEQVPILQIGD-LILAQSQAIVRYLSKKYNICG
 ::.:.. : ..:. ::..:.
632296 MLSSVQFVRINLWKGEHKKPEFLAKNYSGTVPVLELDDGTLIAECTAITEYIDALDGTPT
 50 60 70 80 90 100

**(b)**
 10 20 30 40
PF14_0 MGDNIVLYYFDARGKAELIRLIFAYLGIEYTDKRFGVNGDAFVEFK
 : .....: .: .. .:...: .: . ... ::...
632296 MNGRGFLIYNGGEKMKQKMIIYDTPAGPYPARVRIALA-------EKNM-LSSVQFVRIN
 10 20 30 40 50

50 60 70 80 90
PF14_0 NFKKEKDTP-FEQ------VPILQIGDLIL-AQSQAIVRY---LSKKYNICGESELNEFY
 .: :...: : . ::.:.. : .: :...::. : :. . ...:...:.. .
632296 LWKGEHKKPEFLAKNYSGTVPVLELDDGTLIAECTAITEYIDALDGTPTLTGKTPLEKGV
 60 70 80 90 100 110

100 110 120 130 140
PF14_0 -------ADMIFCGVQDIHYKFNNTNLFKQNETTFLNEDLPKWS-GYFEKLLKKNHTNNN
 :.. .. ..........: .. : ..:.. :. . .:.:. .: ..
632296 IHMMNKRAELELLDPVSVYFHHATPGLGPEVEL-YQNKE---WGLRQRDKALHGMHYFDT
 120 130 140 150 160

150 160 170 180 190 200
PF14_0 --NDKYYFVGNNLTYADLAVFN--LYDDI-ETKYPSSLKNFPLLKAHNEFISNLPNIKNY
 ... :..:.....::..:.. ....: . ..:..... :.:. . ... :..:..
632296 VLRERPYVAGDSFSMADITVIAGLIFAAIVKLQVPEECEA---LRAWYKRMQQRPSVKKL
 170 180 190 200 210 220

210
PF14_0 ITNRKESVY
 .. :..:
632296 LEIRSKSS
 230
