# Supplementary material for: ApicoAlign: an alignment and sequence search tool for apicomplexan proteins
Source: BMC Genomics. 2011 Nov 30;12(Suppl 3):S6. doi: 10.1186/1471-2164-12-S3-S6 (PMC3333189; doi:10.1186/1471-2164-12-S3-S6)
Supplement: Additional file 10 — Supplementary Figure 8: Alignment extension of experimentally characterized P. falciparum kinase with PfFSmat60 matrix The sequences compared are the experimentally characterized P. falciparum protein kinase (PF11_0220) and PIK-related protein kinase and rapamycin target of Saccharomyces cerevisiae (gi: 6322526). While BLOSUM50 gave an alignment score of 23.4 bits at an E-value 0.31 with an overlap of only 71 amino acid residues (637-707:2090-2157), PfFSmat60 gave an alignment score of 4872.5 bits at an E-value 0.0 and the overlap was 1990 amino acid residues (1-1675:375-2307). The fasta program (FASTA package, version 3) was used for alignment. [file 1471-2164-12-S3-S6-S10.pdf]

```
(a)
        610       620       630       640       650       660
PF11_0 MERGEQKQNKKKIYNNNNINSHNNSCNSNIIQKNETKFSESKGNNKNKKFKSEDSNKLEE
                                     : : :  ::  ..... .::. . :.   .
632252 LQHVSPQLLATHDLELAVPGTYFPGKPTIRIAKFEPLFSVISSKQRPRKFSIKGSDG--K
    2060      2070      2080      2090      2100      2110


        670       680       690       700       710       720
PF11_0 NYNIFLKKIKEINYNTLKKSFYNFVYPMFKNENIYNFSEHIMHKRNKKCPVEHIKQHMEN
       .:.  ::  ..:  ..:  .....:  ..::..   :..:.
632252 DYKYVLKGHEDIRQDSLVMQLFGLVNTLLKNDS-ECFKRHLDIQQYPAIPLSPKSGLLGW
      2120      2130      2140      2150       2160      2170


(b)                                            10        20        30
PF11_0                               MSKSLKLMEDKNLEKNAVEAFKQKMEKKKQ
                                     ..: :....:. ::   ..: ..:... :.
632252 IAYENHKAKMIREKIYQIVPLLASFNPQLFAGKYLHQIMDNYLEIL-TNAPANKIPHLKD
          350       360       370       380       390        400

               40        50                 60                  70
PF11_0 EYLRFNLSRNGENMKEGDEKKK---------EIDINNNHNFN----------INNHHVND
       .  .. .: .  . . :.. ..         : :.... .:.          :.. .:.
632252 DKPQILISIGDIAYEVGPDIAPYVKQILDYIEHDLQTKFKFRKKFENEIFYCIGRLAVPL
           410       420       430       440       450       460

              80                 90       100        110       120
PF11_0 KHINNKYINNYMNNRHIK---------NIHVNNDHINNIHVS-NNHINNIHVSNNNHINNN
         . .:..:. . . ..:         .... ...:...  . :... :. :...  ...
632252 GPVLGKLLNRNILDLMFKCPLSDYMQETFQILTERIPSLGPKINDELLNL-VCSTLSGTP
           470       480       490       500       510        520

             130       140       150       160            170
PF11_0 HIKNNNDINRKNSNRHKNRKVVYIETYDTYDKYILNMYDEENEIYED-----EINR--EN
        :....... .. .:...:         . ..: ::.   :.:.  .:      .. :  .:
632252 FIQPGSPMEIPSFSRERAR--------EWRNKNILQKTGESNDDNNDIKIIIQAFRMLKN
            530       540                 550       560       570

          180             190       200       210       220
PF11_0 VESRTPNEGMEFNRI------EKREENIFIPLKILSNKYDFNDLVEATNNFSEYNRIAKG
       ..:: .   .:: ::      :. ....   ... : .  ..: .  .......: ...
632252 IKSRFSL--VEFVRIVALSYIEHTDPRVRKLAALTSCEIYVKDNICKQTSLHSLNTVSEV
          580         590       600       610       620       630
      230       240       250       260             270
PF11_0 GNGTVYKGVLKNCINVAIKVLKKNENNGFENEIIIMSRYR------HN---NILS----L
        .  ..  ........ ..::: : :. :...... . .:       :.   ::.:    :
632252 LSKLLAITIADPLQDIRLEVLK-NLNPCFDPQLAQPDNLRLLFTALHDESFNIQSVAMEL
            640       650        660       670       680       690
         280       290       300       310                   320
PF11_0 LGYATNKNNFYLIYEYVNLGDLRTLLFNHYYYYNSKNKE------------NPELSYYNI
       .:. ...:..:.: .  ..  : .:: .  .. .:..::            . ...   :
632252 VGRLSSVNPAYVIPSIRKI--LLELLTKLKFSTSSREKEETASLLCTLIRSSKDVAKPYI
             700       710         720       730       740
```

```
           330         340                  350       360       370
PF11_0 SCYENYLRKK--KSSSSCSSTNYYT-----------STFYKQNIFPYFNNSKYSPFQQNS
       . . :.: .:  ..:...:...:           ...: ...:.. .. ..  :.::
632252 EPLLNVLLPKFQDTSSTVASTALRTIGELSVVGGEDMKIYLKDLFPLIIKTFQD--QSNS
     750       760       770       780       790       800

                              380                    390       400
PF11_0 FDNNTPL----------------FLSFNIRINILV-------------QIINVLCYLHTS
       :.....:                .:...  ..:::             :.....  : .
632252 FKREAALKALGQLAASSGYVIDPLLDYPELLGILVNILKTENSQNIRRQTVTLIGILGAI
       810       820       830       840       850       860

              410       420       430       440            450
PF11_0 SPIVY-HRDLKSANILIDDQFNAKLGDFGLSFVYMNNNNVFNLTGGTPG-----YADPYY
       .:  . .:...:.. . ..: ::.  :..: .  :...:    :  .       . ::..
632252 DPYRQKEREVTSTTDISTEQ-NAPPIDIALLMQGMSPSNDEYYTTVVIHCLLKILKDPSL
       870       880        890       900       910       920

         460       470       480       490       500       510
PF11_0 ISTHEINEQTEIYSFGALILEMLVSKSPAIHVGKNYNCIYSKNEKCPIFYHKKKHDHDDN
        : :..  :. .. : .: :.. ::    :  .  . ...... ....  :: ..  .
632252 SSYHTAVIQAIMHIFQTLGLKC-VSFLDQI-IPTILDVMRTCSQSLLEFYFQQLCSLIII
        930       940        950        960       970       980

         520       530          540       550       560        570
PF11_0 VFDYLVNHLNMSDYKSIY-SILDYS--VNFPDFLVEKLTKLSFLCLNPNIKNR-PSSKLV
       :  ... .:..     ::. .: :.:  ...   ::......:  .:. ..:.  : .  .
632252 VRQHIRPHVD-----SIFQAIKDFSSVAKLQITLVSVIEAIS-KALEGEFKRLVPLTLTL
          990           1000      1010      1020       1030

             580       590           600              610       620
PF11_0 NLILLEIQKECDLFMKKQQ----ETFKRKFNCIN-------MYLEDMERGEQKQNKKKIY
        :..:: .:..: .....     :.:  ...  .       ........:. ....  .
632252 FLVILENDKSSDKVLSRRVLRLLESFGPNLEGYSHLITPKIVQMAEFTSGNLQRSAIITI
     1040      1050      1060      1070      1080      1090

              630       640       650       660       670       680
PF11_0 NNNNINSHNNSCNSNIIQKNETKFSESKGNNKNKKFKSEDSNKLEENYNIFLKKIKEINY
       ..  .  .  .   ...:.:...  ...:...... .:..... :  : .. . :.  :. ::
632252 GKLAKDVDLFEMSSRIVHSLLRVLSSTTSDELSKVIMNTLSLLLIQMGTSFAIFIPVINE
     1100      1110      1120      1130      1140      1150

              690       700         710        720       730
PF11_0 NTLKKSFYNFVYPMFKNENIYN--FSEHIMHKRNKKC-PVEHIKQHMENAKKVSSTMEYN
         .:.... .:  ..:. . :  ....:... .... :.:.    ::.:..  .... :
632252 VLMKKHIQHTIYDDLTNRILNNDVLPTKILEANTTDYKPAEQ----MEAADAGVAKLPIN
     1160      1170      1180      1190      1200          1210

       740       750         760            770       780
PF11_0 ELNMNDIPTPDYIRFK--YNTISNLCNI---KECILH--RMHKDFFSKNYILNSFSFN-L
       .  .... .....: :  ... :. ..:   ::.  :  :. ....:. : :..  :: .
632252 QSVLKSAWNSSQQRTKEDWQEWSKRLSIQLLKESPSHALRACSNLASMYYPLAKELFNTA
         1220      1230      1240      1250      1260      1270
```

```
       790       800              810       820       830        840
PF11_0 MNCYFLKFISNYLKY------VNMPNGSNSLLIKDGDDDDDDDNEHDEEDYD-DEKEEGE
       ..:.. .. :.:..       .....  :.  :..    .  . .::::..... .... ::
632252 FACVWTELYSQYQEDLIGSLCIALSSPLNPPEIHQTLLNLVEFMEHDDKALPIPTQSLGE
         1280      1290      1300      1310      1320      1330

           850       860       870       880       890       900
PF11_0 Y-EQVHLSKKKYIEDKYKEEIYKEEIYKEEIYKEEIYKEEIYKEEIYKEEIYKEEIYKGE
       : :..:. .:..   .:::  . .:  ...: .. :. ....... ..  :.:..... .
632252 YAERCHAYAKAL---HYKEIKFIKEPENSTI-ESLISINNQLNQTDAAIGILKHAQQHHS
         1340         1350      1360        1370      1380      1390

           910       920          930                   940
PF11_0 IYKEEIYKEEIYKEEKYKDDIH---DEEKYKD-----------DIH--NEEKYKNDIHNE
       .. .:.. :   : :...:..:   ..::. :           ..:  .: .. ......
632252 LQLKETWFE---KLERWEDALHAYNEREKAGDTSVSVTLGKMRSLHALGEWEQLSQLAAR
                  1400      1410      1420      1430      1440

         950        960         970       980          990
PF11_0 ENKEGK-PSSSFKTPIER--LFKIYQEDIHNNMNNKLKKKKTMKE---QIQQKKKNNNIS
       . : .:  .... .:..   .. . . :. ..  ...:.:.. ::   .:   .::.  .
632252 KWKVSKLQTKKLIAPLAAGAAWGLGEWDMLEQYISVMKPKSPDKEFFDAILYLHKNDYDN
      1450      1460      1470      1480      1490      1500


    1000        1010      1020      1030      1040      1050
PF11_0 NINHIDNSNN--VDNNKENVNNKKNKQMNLEENNIYNNNNNNNNADNNQNIYNKNINYH
       . .:: :...  :.. .. .:.. :.. ..  ...  .. ..  . .. .. ..:...:.
632252 ASKHILNARDLLVTEISALINESYNRAYSVIVRTQIITEFEEIIKYKQLPPNSEKKLHYQ
      1510      1520      1530      1540      1550      1560
      1060      1070      1080      1090      1100       1110
PF11_0 NNISNNPFKGIEKDYIDKNNMINVIPKCPLKNPNLNNIYANYAN-NQNDVRYQVPFNNND
       :  ... . :..:. .:  . .  . .. .:. .  .:. ..::  ... :.... .. .
632252 NLWTKR-LLGCQKN-VDLWQRVLRVRSLVIKPKQDLQIWIKFANLCRKSGRMRLANKALN
      1570       1580       1590      1600      1610      1620

       1120            1130      1140      1150      1160
PF11_0 YLLNNHN-------FLEPNPDHINNINNNNNNNYYYYYNNNQIYMNNCYMNN---KYNNM
       .:.. :        : .:.:   ....   ....:  . :. : ... ....   . :::
632252 MLLEGGNDPSLPNTFKAPPPVVYAQLKYIWATGAYKEALNHLIGFTSRLAHDLGLDPNNM
        1630      1640      1650      1660      1670      1680
       1170        1180      1190      1200       1210      1220
PF11_0 NMEHMK--NYYNNGYNNEYYNHINLSNIPYNNIQLQNGHFH-NINMNNQNMIPFQNINNT
        ....:  ..... : .:: . ..         .. :..:... ... : .:. : . ...
632252 IAQSVKLSSASTAPYVEEYTKLLA-------RCFLKQGEWRIATQPNWRNTNPDAILGSY
        1690      1700              1710      1720      1730

          1230      1240        1250       1260      1270
PF11_0 LYASNQVKINNEHIINDVHNN--ICNNMQNISK-NVGKSSSSDINHEINDAQNI---LSN
       :.:..  :   ..  : . .:  .....:. .: : ::....: ..  ::  .:   . .
632252 LLATHFDKNWYKAWHNWALANFEVISMVQEETKLNGGKNDDDDDTAVNNDNVRIDGSILG
     1740      1750      1760      1770      1780      1790

      1280            1290        1300                    1310
PF11_0 SNEQYLKGNY-------RNNTPKIS--FHLNK--EKNNEQD------------IKEACL
```

```
       :..  ..::.       :. .:.:.  ::  .  :..  ::             :::...
632252 SGSLTINGNRYPLELIQRHVVPAIKGFFHSISLLETSCLQDTLRLLTLLFNFGGIKEVSQ
     1800      1810      1820      1830      1840      1850

               1320      1330       1340
PF11_0 NNKEN-----ISKNLENLRKLHNDI-QKEKDVNN------------------------IK
       .. :.     :.. :: : .: ..: :....:.:                        ::
632252 AMYEGFNLMKIENWLEVLPQLISRIHQPDPTVSNSLLSLLSDLGKAHPQALVYPLTVAIK
     1860      1870      1880      1890      1900      1910

          1350      1360      1370      1380             1390
PF11_0 ETDEDTYCNSSSLLRDYNIHKKNMNNNDLLLSDDNFMMSP------YDNIND-THILYIS
       ... ... .. :.. . .::.. . :.. :.:.. . ..       :....: .......
632252 SESVSRQKAALSIIEKIRIHSPVLVNQAELVSHELIRVAVLWHELWYEGLEDASRQFFVE
     1920      1930      1940      1950      1960      1970

       1400         1410         1420      1430            1440
PF11_0 NFVN--FINLEN-NKNI-NDSNTCA--WFNKNVFEVLIDEHKFFSS------VTFLDILY
       . ..  : .::. .:.. :...:..   :.:.  . : :.......      .. :....
632252 HNIEKMFSTLEPLHKHLGNEPQTLSEVSFQKSFGRDLNDAYEWLNNYKKSKDINNLNQAW
     1980      1990      2000      2010      2020      2030

           1450                    1460      1470      1480
PF11_0 E--FNVFN-VKRD---------GHV----FIEHDNEYVLQGKWCDYDPNKNNSTSVNFEE
       .  .:::. ..:.          ::    ...:: :....:.   : :.:.. . ..::.
632252 DIYYNVFRKITRQIPQLQTLDLQHVSPQLLATHDLELAVPGT---YFPGKPTIRIAKFEP
     2040      2050      2060      2070      2080         2090

     1490      1500      1510      1520      1530      1540
PF11_0 NFCFEIAISEDNENNTHSVFLLKSNQLKYTDNIQNDNTITKKIWVSEYLGRRNEFLE---
        :.   .::....... :.    :.  .:.  ......:...  :....:  :..:.
632252 LFS---VISSKQRPRKFSI--KGSDGKDYKYVLKGHEDIRQDSLVMQLFGLVNTLLKNDS
           2100      2110        2120      2130      2140      2150

        1550      1560               1570          1580      1590
PF11_0 KILKRNISNILYECI--SRKHCLF-------LLEVLMKKINK----PTSYDNKVKGNFSY
       ...::... ..:..:  : :. :.        ..::... ..    : . .. :. ...
632252 ECFKRHLDIQQYPAIPLSPKSGLLGWVPNSDTFHVLIREHRDAKKIPLNIEHWVMLQMAP
             2160      2170      2180      2190      2200      2210

           1600      1610                  1620      1630      1640
PF11_0 FYFEYNIKVLCNSENCIF----TNNHILHK--------NNTYSYKLPNNILSLLVFSEDK
        :   :...: . :  ..    :... :.:        ..:.  .  . . ::.:.:..
632252 DY--ENLTLLQKIEVFTYALDNTKGQDLYKILWLKSRSSETWLERRTTYTRSLAVMSMTG
               2220      2230      2240      2250      2260

               1650         1660      1670
PF11_0 LIKG--KYHPENQI--NTTQKV-HFPLYPFFVNINFYNEK
        : :  ..::.: .  . : :: :. .   : ......::
632252 YILGLGDRHPSNLMLDRITGKVIHIDFGDCF-EAAILREKYPEKVPFRLTRMLTYAMEVS
     2270      2280      2290       2300      2310      2320
```

Word to PDF
