# Supplementary material for: ApicoAlign: an alignment and sequence search tool for apicomplexan proteins
Source: BMC Genomics. 2011 Nov 30;12(Suppl 3):S6. doi: 10.1186/1471-2164-12-S3-S6 (PMC3333189; doi:10.1186/1471-2164-12-S3-S6)
Supplement: Additional file 11 — Supplementary Table S5: Pairwise alignments of Cyptosporidium parvum ACBP1 (cgd1_1140) against Arabidopsis thaliana ACBP1 (gi:15238757) The pairwise alignment was performed between two experimentally characterized proteins Cyptosporidium parvum ACBP1 (cgd1_1140) and Arabidopsis thaliana ACBP1 (gi:15238757) using water program (EMBOSS package) and FASTA program. [file 1471-2164-12-S3-S6-S11.doc]

**Supplementary Table S5: Pairwise alignments of *Cyptosporidium parvum* ACBP1 (cgd1_1140) against *Arabidopsis thaliana* ACBP1 (gi:**15238757).

| **fasta program (FASTA package, version 3.0)** | | | | |
| --- | --- | --- | --- | --- |
| **Matrix used** | **bits score & E-value** | **Overlap length with residue position** | | |
| BLOSUM100 | 70.5 & 5.3e-17 | 237 aa ( 26-259:118-327) | | |
| PAM2  PfFSmat60 | 7.4 & 1.0  378.9 & 7.8e-110 | 14 aa ( 26-38:118-130)  274 aa ( 1-268:94:337) | | |
| **water program (EMBOSS package, version 6.3.1)**   | **Matrix used** | **Score, identity (%) & similarity(%)** | **alignment length** | | --- | --- | --- | | | | |  |
| EBLOSUM62 | 298.5, 33.1 & 49.2 | | 254 aa |  |
| EBLOSUM90 | 289.5, 32.4 & 44.7 | | 262 aa |  |
| EBLOSUM50  EPAM200  PfFSmat60 | 411, 31.9 & 48.9  369.5, 31.6 & 53.6  945, 30.3% & 63.5 | | 270 aa  263 aa  304 aa |  |

Note: The query start & end and subject start & end for the alignment overlap respectively are provided in the brackets under column 3 for fasta program.
