# Supplementary material for: ApicoAlign: an alignment and sequence search tool for apicomplexan proteins
Source: BMC Genomics. 2011 Nov 30;12(Suppl 3):S6. doi: 10.1186/1471-2164-12-S3-S6 (PMC3333189; doi:10.1186/1471-2164-12-S3-S6)
Supplement: Additional file 12 — Supplementary Figure 9: Alignment extension of probable P. chabaudi bi-functional enzyme of the shikimate pathway The sequences compared here are the P. chabaudi hypothetical protein, PCHAS_041350 and yeast multifunctional protein, Aro1p (gi:6320332). (a) The alignment with BLOSUM50 showing the aligned motif regions for only EPSP synthase I motif (gray shading). (b) The alignment extended by PfFSmat60 for both the EPSP synthase I and shikimate kinase motifs represented as (i) and (ii) respectively. The fasta program (FASTA package, version 3) was used for alignment. [file 1471-2164-12-S3-S6-S12.doc]

**(a)**
 590 600 610 620 630 640
PCHAS_ FQDKIIFNVYNSGTVCRLILPLLCLYICKQNLKAKKENKTLLKFIVLKGNKQMESVRIIS
 .: : .. . :.::: :: .:.. : :.
632033 VVEGHGGSTLSACADPLYLGNAGTASRFLTSLAALVNSTSSQKYIVLTGNARMQQ-RPIA
 480 490 500 510 520 530

**(b)** (i)

540 550 560 570 580 590
PCHAS_ YKSIFDKIDKGHIATYKCKQNEYHLSITGNIDNRNSMFLFKNFIFQDKIIFNVYNSGTVC
 ...... :: :: . ..: .. ..:. . :. .:.. .. :.::..
632033 LTAVHEL--KG--ATISWEDNGETVVVEGHGGSTLSAC-------ADPLYLG--NAGTAS
 460 470 480 490 500

600 610 620 630 640 650
PCHAS_ RLILPLLCLYICKQNLKAKKENKTLLKFIVLKGNKQMESVRIISPLVKVIQKSFKYIKII
 :.. ..:.: ..... .:.:::.::..:.. : :.:::. .... . ::
632033 RFL----------TSLAALVNSTSSQKYIVLTGNARMQQ-RPIAPLVDSLRANGT--KIE
 510 520 530 540 550

(ii)

1070 1080 1090 1100 1110 1120
PCHAS_ RKLDKIFEKRKREMGKAGSTREPNNKIKYHVFEKIHINYYFNDINKLLTKVVIDAEYFSD
 .::..::... :...:. :.:. . . :.. .. . : :: . ..:
632033 -DLDELFEQQHN-----------NQSVKQFVVENGWEK--FREEETRIFKEVI--QNYGD
 920 930 940 950 960

1130 1140 1150 1160 1170 1180
PCHAS_ DFFPICVLFCYYLIIHKSENILFKIKNIHNQDIKESIRINNVVYILKMCFQNLVFISCD-
 : . :. . :....:. :.. .:...: . :... . . ...::...:
632033 DGY---VFSTGGGIVESAES----RKAL--KDFASSG--GYVLHLHRDIEETIVFLQSDP
 970 980 990 1000 1010
