# Supplementary material for: ApicoAlign: an alignment and sequence search tool for apicomplexan proteins
Source: BMC Genomics. 2011 Nov 30;12(Suppl 3):S6. doi: 10.1186/1471-2164-12-S3-S6 (PMC3333189; doi:10.1186/1471-2164-12-S3-S6)
Supplement: Additional file 13 — Supplementary Figure 10: Alignment extension of probable P. knowlesi bi-functional enzyme of the shikimate pathway The sequences compared here are the P. knowlesi hypothetical protein, PKH_041350 and yeast multifunctional protein, Aro1p (gi:6320332). (a) The alignment with BLOSUM50 showing the aligned motif regions for only EPSP synthase I motif (gray shading). (b) The alignment extended by PfFSmat60 for both the EPSP synthase I and shikimate kinase motifs represented as (i) and (ii) respectively. The fasta program (FASTA package, version 3) was used for alignment. [file 1471-2164-12-S3-S6-S13.doc]

**(a)**

580 590 600 610 620 630
PKH_04 YILHIKGNIQRSVFLFKRFIYERGITLNVYNCGTVCRFILPLLCIYICKQNLKAKKRKKK
 .. ..:. .. .: . : ::. ::. .: : . .
632033 TVV-VEGHGGSTLSACADPLY-------LGNAGTASRFLT----------SLAALVNSTS
 480 490 500 510

**(b)** (i)

850 860 870 880 890 900
PKH_04 FSTTSKSFIDLTIRVMKLWGVQVGMKNFCYVLKKSGRYPSYGRGRRELALRKFLVSRIKR
 ..: . : : .... . . : ..: ...: .::. . .:..:
632033 NGET--------VVVEGHGGSTLSACADPLYLGNAG---TASRFLTSLAALVNSTSSQK-
 480 490 500 510 520

(ii)

1380 1390 1400 1410 1420 1430
PKH_04 YPPSAEPRPEEGPPSEASLMWSDSSARSDIHDGSTEMENFPRGSQVK--CIQN-YQK-RG
 . .: : . .. :..:. .. : .. .:. ..:: ...: ..: :
632033 R--AA------GKTTISK--WCASALGYKLVDLDELFEQQHNNQSVKQFVVENGWEKFRE
 900 910 920 930 940

1440 1450 1460 1470 1480 1490
PKH_04 EKNMAIKMGEAEKEKNFHVDMEMWPNIKEGTRREGTKFTDVQTENPPSGGDGSTNSSLHL
 :.. .: :. .:. : .... : :... ... .. ..::: :::
632033 EETRIFK----EVIQNYGDDGYVFST--GGGIVESAESRKALKDFASSGGY-----VLHL
 950 960 970 980 990
