# Supplementary material for: ApicoAlign: an alignment and sequence search tool for apicomplexan proteins
Source: BMC Genomics. 2011 Nov 30;12(Suppl 3):S6. doi: 10.1186/1471-2164-12-S3-S6 (PMC3333189; doi:10.1186/1471-2164-12-S3-S6)
Supplement: Additional file 14 — Supplementary Figure 11: Alignment extension of probable P. vivax bi-functional enzyme of the shikimate pathway The sequences compared here are the P. vivax hypothetical protein, PVX_003750 and yeast multifunctional protein, Aro1p (gi:6320332). (a) The alignment with BLOSUM50 showing the aligned motif regions for only EPSP synthase I motif (gray shading). (b) The alignment extended by PfFSmat60 for both the EPSP synthase I and shikimate kinase motifs represented as (i) and (ii) respectively. The fasta program (FASTA package, version 3) was used for alignment. [file 1471-2164-12-S3-S6-S14.doc]

**(a)**

570 580 590 600 610 620
PVX_00 SPGGNVLHIRGNIQRSAFLFKRFTYRRGITLNVYNCGTVCRFILPLLCLYICKQNLKAKR
 : . : ::. ::. : : : ...
632033 KGATISWEDNGETVVVEGHGGSTLSACADPLYLGNAGTASRFLTSLAALV----NSTSSQ
 470 480 490 500 510

**(b)** (i)

580 590 600 610 620 630
PVX_00 NVLHIRGNIQRSAFLFKRFTYRRGITLNVYNCGTVCRFILPLLCLYICKQNLKAKRRKKR
 ..: :: . .: . :.::..::. .:..:. .....:
632033 STL--------SAC---------ADPLYLGNAGTASRFLTSLAALVNSTSSQK-------
 490 500 510 520

(ii)

1160 1170 1180 1190 1200 1210
PVX_00 RVYNAVIILK----LFFQNVLFISCDRNSVYIDRMTHPVENCLFYRMSKKGELPPGEAPS
 : :. .. .:..:. : . :. . ::.. :.: : .
632033 -VENGWEKFREEETRIFKEVIQNYGDDGYVFSTG-GGIVESA----ESRK-------ALK
 940 950 960 970 980
