# Supplementary material for: ApicoAlign: an alignment and sequence search tool for apicomplexan proteins
Source: BMC Genomics. 2011 Nov 30;12(Suppl 3):S6. doi: 10.1186/1471-2164-12-S3-S6 (PMC3333189; doi:10.1186/1471-2164-12-S3-S6)
Supplement: Additional file 15 — Supplementary Figure 12: Alignment extension of probable P. yoelii bi-functional enzyme of the shikimate pathway The sequences compared here are the P. yoelii hypothetical protein, PY00069 and yeast multifunctional protein, Aro1p (gi:6320332). (a) The alignment with BLOSUM50 showing the aligned motif regions for only EPSP synthase I motif (gray shading). (b) The alignment extended by PfFSmat60 for both the EPSP synthase I and shikimate kinase motifs represented as (i) and (ii) respectively. The fasta program (FASTA package, version 3) was used for alignment. [file 1471-2164-12-S3-S6-S15.doc]

(a)

730 740 750 760 770 780
PY0006 NKKINDLYKNTKYKIDKLNNNYVNNISIILGGGIIEFENSRHIIKKLKN----VIIIKRD
 ::::.: .::. .: . . :. ..::
632033 NGWEKFREEETRIFKEVIQNYGDDGYVFSTGGGIVESAESRKALKDFASSGGYVLHLHRD
 940 950 960 970 980 990

790 800 810 820 830 840
PY0006 ENELYEICIHDKIKPKLSGNLNKIIKRRTILFNELNIPFHFSIPTEYIINKNIKKLNKTR
 .: . : .: .. .. .:: ..: . : : :
632033 IEETIVFLQSDPSRPAYVEEIREVWNRREGWYKECS-NFSFFAPHCSAEAEFQALRRSFS
 1000 1010 1020 1030 1040 1050

(b) (i)

300 310 320 330 340
PY0006 IKESIRINNVVYILK--LVFQNFVFILRDKNNIYITRFRNYIDNCIFNLNSNKNYI---D
 :. . ..::. . .... . : :. ..:: ... ....: .:...::
632033 ISWEDNGETVVVEGHGGSTLSACADPLYLGNAGTASRFLTSL-AALVNSTSSQKYIVLTG
 470 480 490 500 510 520

(ii)

720 730 740 750 760 770
PY0006 VNNDDVIFYNKKINDLYKNTKYKIDKLNNNYVNNISIILGGGIIEFENSRHIIKKLKN--
 :.: :...... ..:.. .. ...:: .. ::::.: ..::...:....
632033 VENGWEKFREEETR-IFKEVIQNYG--DDGYVFST----GGGIVESAESRKALKDFASSG
 940 950 960 970 980
