# Supplementary material for: ApicoAlign: an alignment and sequence search tool for apicomplexan proteins
Source: BMC Genomics. 2011 Nov 30;12(Suppl 3):S6. doi: 10.1186/1471-2164-12-S3-S6 (PMC3333189; doi:10.1186/1471-2164-12-S3-S6)
Supplement: Additional file 16 — Supplementary Table S4: Pairwise alignments of probable plasmodia bifunctional proteins of shikimate pathway with yeast AROM complex The pairwise alignments of probable plasmodia bifunctional proteins of shikimate pathway with yeast AROM complex using water program (EMBOSS package) and FASTA program. [file 1471-2164-12-S3-S6-S16.doc]

## Supplementary Table S4 - Pairwise alignments of probable plasmodia bifunctional proteins of shikimate pathway with yeast AROM complex

| **EMBOSS/water program** | **BLOSUM62 matrix** | | **PfFSmat60 matrix** | |
| --- | --- | --- | --- | --- |
| Organism | Length | Similarity (%) | Length | Similarity (%) |
| *P. berghei* (PBANKA_030400)  *P. chabaudi* (PCHAS_030620)  *P. falciparum* (PFB0280w)  *P. knowlesi* (PKH_041350)  *P. vivax* (PVX_003750)  *P. yoelii* (PY00069) | 601  2045  1288  1589  2022  889 | 35.30  29.70  33.60  31.40  28.10  29.80 | 2205  2101  2332  2334  2349  1557 | 49.10  50.10  46.00  46.60  43.90  47.70 |
|  |  |  |  |  |
| **FASTA program** | **BLOSUM50 matrix** | | **PfFSmat60 matrix** | |
| Organism | Length | E-value | Length | E-value |
| *P. berghei* (PBANKA_030400)  *P. chabaudi* (PCHAS_030620)  *P. falciparum* (PFB0280w)  *P. knowlesi* (PKH_041350)  *P. vivax* (PVX_003750)  *P. yoelii* (PY00069) | 110  109  131  129  129  74 | 2.20E-005  2.00E-018  2.00E-012  0.0065  3.40E-008  4.40E-006 | 1772  1930  1781  1834  1964  1206 | 4.50E-108  0  0  1.90E-189  0  3.00E-198 |
