# Supplementary material for: ApicoAlign: an alignment and sequence search tool for apicomplexan proteins
Source: BMC Genomics. 2011 Nov 30;12(Suppl 3):S6. doi: 10.1186/1471-2164-12-S3-S6 (PMC3333189; doi:10.1186/1471-2164-12-S3-S6)
Supplement: Additional file 17 — Supplementary Table S3: Pairwise alignments of MAL7P1.156 against four known yeast triglyceride lipases The table shows the E-values, bits scores and alignment length of pairwise alignments between probable P. falciparum acyl glycerol lipase (MAL7P1.156) and four known yeast triglyceride lipases (tgl2p, tgl3p, tgl4p and tgl5p). [file 1471-2164-12-S3-S6-S17.doc]

Supplementary Table S3: Pairwise alignments of MAL7P1.156 against four known yeast triglyceride lipases

| **Subject** | **Matrix used** | **bits score & E-value** | **Overlap length with residue position** |
| --- | --- | --- | --- |
| tgl2p (gi:6320263) | BLOSUM50 | 14.0 & 1.0 | 97 aa (456-547:50-144) |
| tgl3p (gi:6323973) | BLOSUM50 | 19.7 & 0.43 | 105 aa (345-449:363-458) |
| tgl4p (gi:6322942) | BLOSUM50 | 17.8 & 0.94 | 160 aa (341-497:51-205) |
| tgl5p (gi:6324655) | BLOSUM50 | 21.6 & 0.15 | 30 aa (212-241:370-395) |
| tgl2p (gi:6320263) | BLOSUM100 | 12.2 & 1.0 | 8 aa (513-520:156-163) |
| tgl3p (gi:6323973) | BLOSUM100 | 7.4 & 1.0 | 9 aa (268-276:511-519) |
| tgl4p (gi:6322942) | BLOSUM100 | 13.7 & 1.0 | 13 aa (585-597:517-529) |
| tgl5p (gi:6324655) | BLOSUM100 | 12.3 & 1.0 | 15 aa (212-226:370-384) |
| tgl2p (gi:6320263) | PAM2 | 8.1 & 1.0 | 10 aa (625-630:155-164) |
| tgl3p (gi:6323973) | PAM2 | 10.3 & 1.0 | 4 aa (421-424:266-269) |
| tgl4p (gi:6322942) | PAM2 | 10.2 & 1.0 | 4 aa (616-619:101-104) |
| tgl5p (gi:6324655) | PAM2 | 12.9 & 1.0 | 4 aa (554-557:163-166) |
| tgl2p (gi:6320263) | PfFSmat60 | 270.9 & 6.8e-77 | 359 aa (246-596:1-322) |
| tgl3p (gi:6323973) | PfFSmat60 | 390.6 & 1.2e-122 | 682 aa (70-719:5-629) |
| tgl4p (gi:6322942) | PfFSmat60 | 171.7 & 1.3e-46 | 813 aa (4-717:51-802) |
| tgl5p (gi:6324655) | PfFSmat60 | 534.2 & 8.4e-156 | 761 aa (3-714:36-746) |

Note: The query start & end and subject start & end for the alignment overlap respectively are provided in the brackets under column 4.
