# Supplementary material for: ApicoAlign: an alignment and sequence search tool for apicomplexan proteins
Source: BMC Genomics. 2011 Nov 30;12(Suppl 3):S6. doi: 10.1186/1471-2164-12-S3-S6 (PMC3333189; doi:10.1186/1471-2164-12-S3-S6)
Supplement: Additional file 18 — Supplementary Figure 13: Alignment of probable missing enzyme of P. falciparum glycerol biosynthesis pathway The sequences compared are conserved protein in Plasmodium sps. with unknown function, MAL7P1.156 and yeast triacylglycerol lipase tgl5p which has patatin domain for lipase activity spanning 183 to 388 residues. (a) The alignment with BLOSUM50 covered only few residues of patatin domain (grey shaded). (b) The alignment generated with PfFSmat60 covered whole patatin domain of subject sequence. The fasta program (FASTA package, version 3) was used for alignment. [file 1471-2164-12-S3-S6-S18.doc]

a)

190 200 210 220 230 240

psu|MA HSPYISIEKLVEDYFTYSSYFIENIYDNYKNLSFLSNNTDSDIPILLIHGKEDEIIHVSH

:..:.....:.:.:: .. .:...:.:

gi|632 PGVFPSTPLFEKDPHTGKIKEWGATNLHLSNMKFMDGSVDNDMPI----SRLSEMFNVDH

340 350 360 370 380 390

250 260 270 280 290 300

psu|MA SEYLMKNLNNKFKHASYPTDSYHNYYYVIDDLGIPIKIFLETLSKSKNAKSVDINIPKVY

gi|632 IIACQVNIHVFPLLKFSNTCVGGEIEKEITARFRNQVTKIFKFFSDETIHFLDILKELEF

400 410 420 430 440 450

b)

140 150 160 170 180 190

psu|MA ALAAYNFLKSLNIKNENILLFGRSIGTGVASKLAYNL--KLIGVSVAGIILHSPYISIEK

::. .. .... :: .::. .:. . ..: :.:. : ::.:..: ..

gi|632 ALVLSG--------GSTFGLF--HIGV-LAALFESDLMPKVISGSSAGAIVASIFC----

190 200 210 220

200 210 220 230 240

psu|MA LVEDYFTYSSYFIENIYDNYKNLSF-LSNNTDSDIPILLIHGKEDEIIHVSHSEYLMKNL

..... :.....:. :..: . :...:. : .:. .:..:. . . .

gi|632 ------VHTTQEIPSLLTNVLNMEFNIFNDDNSKSP------NENLLIKISRFCQNGTWF

230 240 250 260 270

250 260 270 280 290 300

psu|MA NNK-FKHASYPTDSYHNYYYVIDDLGIPIKIFLETLSKSKNAKSVD-INIPKVYFYRELI

::. . ..... . ... . .. : ..: ....: ...: .. ...:.:.......

gi|632 NNQPLINTMLSFLGNLTFREAYNKTGKILNITVSPASIYEQPKLLNNLTAPNVLIWSAVC

280 290 300 310 320 330

310 320 330 340 350 360

psu|MA YYRINGLDETFKRLNEDEKNKNTNDKKTTKGESSKDICSIDKLTKKRD-----SKKKDKK

.. .:. .:.. . ::...:.. :. :... ...... .. . : :. ...

gi|632 AS--CSLPGVFPSTPLFEKDPHTGKIKE-WGATNLHLSNMKFMDGSVDNDMPISRLSEMF

340 350 360 370 380 390
